# Potential value of PRKDC as a therapeutic target and prognostic biomarker in pan-cancer

**DOI:** 10.1097/MD.0000000000029628

**Published:** 2022-07-08

**Authors:** Xiawei Yang, Feng Yang, Liugen Lan, Ning Wen, Haibin Li, Xuyong Sun

**Affiliations:** a Guangxi Medical University, Nanning, Guangxi Zhuang Autonomous Region, China; b Department of Gynocology, The Second Affiliated Hospital of Guangxi Medical University, Nanning, Guangxi Zhuang Autonomous Region, China; c Transplant Medical Center, The Second Affiliated Hospital of Guangxi Medical University, Nanning, Guangxi Zhuang Autonomous Region, China; d Guangxi Key Laboratory of Organ Donation and Transplantation, Nanning, Guangxi Zhuang Autonomous Region, China; e Guangxi Key Laboratory for Transplantation Medicine, Nanning, Guangxi Zhuang Autonomous Region, China; f Guangxi Transplantation Medicine Research Center of Engineering Technology, Nanning, Guangxi Zhuang Autonomous Region, China.

**Keywords:** DNA-PKcs, genomic alteration, immune infiltration, pan-cancer, PRKDC, prognosis, protein phosphorylation, tumor microenvironment

## Abstract

**Background:**

While protein kinase, DNA-activated, catalytic subunit (PRKDC) plays an important role in double-strand break repair to retain genomic stability, there is still no pan-cancer analysis based on large clinical information on the relationship between PRKDC and different tumors. For the first time, this research used numerous databases to perform a pan-cancer review for PRKDC to explore the possible mechanism of PRKDC in the etiology and outcomes in various tumors.

**Methods:**

PRKDC’s expression profile and prognostic significance in pan-cancer were investigated based on various databases and online platforms, including TIMER2, GEPIA2, cBioPortal, CPTAC, and SangerBox. We applied the TIMER to identified the interlink of PRKDC and the immune infiltration in assorted tumors, and the SangerBox online platform was adopted to find out the relevance between PRKDC and immune checkpoint genes, tumor mutation burden, and microsatellite instability in tumors. GeneMANIA tool was employed to create a protein–protein interaction analysis, gene set enrichment analysis was conducted to performed gene enrichment analysis.

**Results:**

Overall, tumor tissue presented a higher degree of PRKDC expression than adjacent normal tissue. Meanwhile, patients with high PRKDC expression have a worse prognosis. PRKDC mutations were present in almost all The Cancer Genome Atlas tumors and might lead to a better survival prognosis. The PRKDC expression level was shown a positive correlation with tumor-infiltrating immune cells. PRKDC high expression cohorts were enriched in “cell cycle” “oocyte meiosis” and “RNA-degradation” signaling pathways.

**Conclusions:**

This study revealed the potential value of PRKDC in tumor immunology and as a therapeutic target and prognostic biomarker in pan-cancer.

## 1. Introduction

Cells encounter many DNA lesions every day that imperil their genomic completeness, and the most toxic one is double-strand break (DSB). As we all know, one unrepaired DSB might induce cell death, rather a misrepaired DSB can lead to chromosomal abnormalities (e.g., deletions, translocations, and fusions), which may contribute to a loss of heterozygosity, induce genetic instability, neoplastic transformation, and ultimately, cancer.

Organisms have developed wrought genomic stability maintenance systems to prevent cancer^[1]^ by identifying broken DNA sites and restoring the DNA damage.^[2]^ In human cells, DSB repair is mediated by 2 distinguished ways: nonhomologous final junction (NHFJ) and homologous recombination (HR), and NHEJ was considered quicker and more efficient than HR.^[3]^

DNA-dependent protein kinase catalytic subunit (DNA-PKcs) or serine/threonine protein kinase catalytic subunit are the other names for PRKDC. It belongs to the phosphatidylinositol 3-kinase-like (PIKK) family and presents at approximately all mammalian cells.^[4]^ PRKDC interacts with the Ku70/Ku80 heterodimer, then together join into the ligation step of the NHEJ process and, ultimately, helps to preserve genomic integrity.^[5]^

PRKDC has recently attracted a lot of interest in being a therapeutic target and promising biomarker for many human cancers,^[6–13]^ but a pan-cancer investigation of the correlation between PRKDC with various types of tumors based on these large-scale clinical data is currently lacking. In this study, for the first time, we conducted a pan-cancer analysis of PRKDC based on a variety of databases and online platforms to investigate the latent molecular processes of PRKDC in different tumor’s pathogenesis and clinical outcomes.

## 2. Materials and Methods

### 2.1. PRKDC expression analysis

By using Tumor Immune Estimation Resource, version 2 (TIMER2.0, http://timer.cistrome.org/) database,^[14]^ the expression levels of PRKDC in tumor tissues compared with neighboring normal tissue were analyzed in all The Cancer Genome Atlas (TCGA, https://www.cancer.gov/) tumors. For those tumors without normal tissues, we used the Gene Expression Profiling Interactive Analysis, version 2 (GEPIA 2, http://gepia2.cancer-pku.cn)^[15]^ to generate boxplots from the Genotype-Tissue Expression (GTEx, https://gtexportal.org/home/) database, and selected “Match TCGA normal and GTEx data” pattern. This study furthermore generated violin plots of PRKDC expression according to pathological stages of all TCGA tumors by using the “Pathological Stage Plot” modular in GEPIA 2, with cutoff values set to *P* value = .01 and log2 FC (fold change) = 1. Transcripts per million (TPM) values were transformed into a log2 (TPM + 1).

The UALCAN (http://ualcan.path.uab.edu/)^[16]^ enabled us to carried out phosphorylation investigations of PRKDC protein across various tumors from the Clinical proteomic tumor analysis consortium (CPTAC) database.^[17]^ This work compared PRKDC total protein and phosphoprotein expression levels in 6 tumors and neighbor normal tissues, including breast cancer, ovarian cancer, colon cancer, clear cell renal cell carcinoma (RCC), uterine corpus endometrial carcinoma (UCEC), and lung adenocarcinoma (LUAD).

### 2.2. Survival analysis

Connection between PRKDC expression and survival of all TCGA cancers was investigated using “Survival Map” and “Survival Analysis” modules of GEPIA 2. To separate the high and low PRKDC expression groups, a 50% cutoff value was chosen. SangerBox online website (http://sangerbox.com) is a comprehensive Chinese bioinformatics analysis platform. The “Gene-KM plotter” module of SangerBox was used to compare the overall survival (OS), disease-free interval (DFI), disease-specific survival (DSS), and progression-free interval (PFI) in high and low expression cohorts. Hazard ratios (HRs) were computed using a 95% confidence interval and a *P* value.

### 2.3. Genetic alteration analysis

We acquired PRKDC mutation status in tumor patients from the cBioPortal (http://www.cbioportal.org/) platform.^[18]^ The “Cancer Types Summary” section displayed the frequency of alteration, types of mutation, and copy number alteration data. The “Mutations” module presented the mutation site information in the PRKDC protein structural schematic diagram. Using the “Comparison/Survival” module, Kaplan–Meier plots were created to compare DSS, OS, DFS, and PFS in cohorts with or without PRKDC genetic mutation, with log-rank *P* values.

### 2.4. Immunological analysis

To observe the interaction between tumor cells and immune cells, the Tumor Immune Estimation Resource (TIMER, https://cistrome.shinyapps.io/timer/) database^[19]^ was employed. By using “Gene” module of TIMER, we determined the tumors’ purity and discovered the relationship between PRKDC and the quantity of 6 tumor-infiltrating immune cells (TIICs) subsets in 39 kinds of tumors with Spearman correlations. CD4+ T cells, CD8+ T cells, B cells, neutrophils, dendritic cells (DCs), and macrophages are among these 6 categories of TIICs. The correlations between PRKDC and 28 subtypes of immune cell were determined and evaluated via the “Gene-Immune Analysis” module of SangerBox. We also looked at the relational between PRKDC and 47 kinds of gene markers of TIICs in distinct tumors.^[20]^

The all somatic nonsynonymous mutation counts per megabase in coding sequence were used to calculate the tumor mutation burden (TMB), which may be a promising marker for predicting immunotherapy response.^[21]^ Microsatellite instability (MSI) is a molecular tumor characteristic characterized by spontaneous nucleotide loss or gain on short tandem repetitive DNA sequences, and is the result of a defective DNA mismatch repair (MMR) system.^[22]^ Analysis and visualization of TMB and MSI in different tumors were carried out using SangerBox online platform.

### 2.5. Protein–protein interaction network and enrichment analysis

We built a protein–protein interaction (PPI) network for PRKDC using the GeneMANIA online platform (https://genemania.org/)^[23]^ to find out the mechanism of PRKDC in tumorigenesis. The first 5 terms of KEGG (Kyoto encyclopedia of genes and genomes) and HALLMARK analyses were displayed in the high/low PRKDC expression cohorts, respectively, to discover the biological signaling pathway. |NES| > 1, *P* < .01, and FDR < 0.25 were defined as significant gene sets.^[24]^

## 3. Results

### 3.1. PRKDC expression analysis results

**Figure 1.**
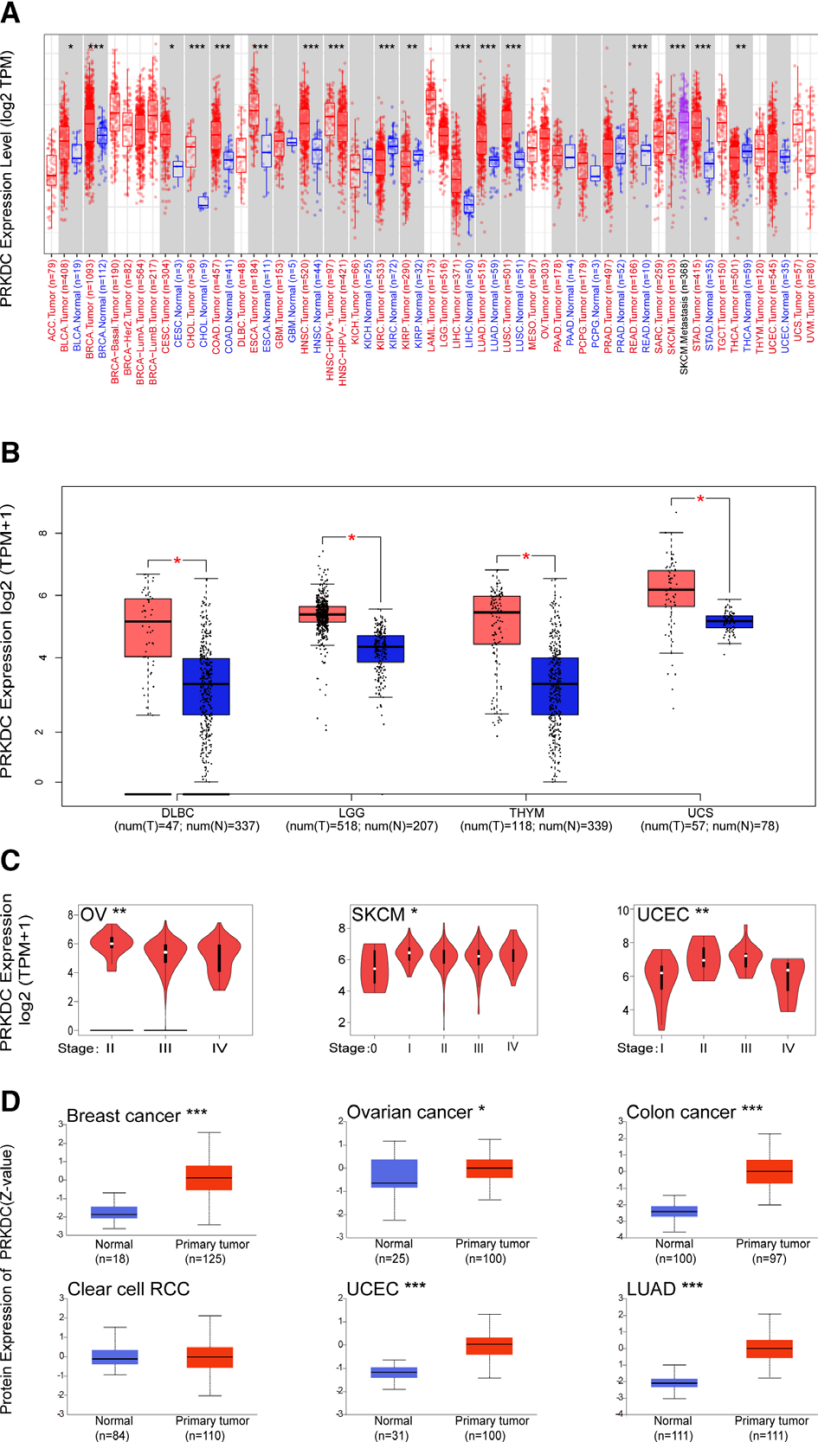
PRKDC expression in different cancers and pathological stages. (A) Comparison of PRKDC expression level in 33 tumor types and normal tissues determined by TIMER2. (B) For those tumors without normal tissues in TCGA database, we used GEPIA 2 to obtain box plots from the GTEx database, and positive results (DLBC, LGG, THYM, and UCS) were shown. T: tumor, N: normal. (C) Violin plots from GEPIA 2 confirmed a positive association between PRKDC expression and advanced cancer stages in OV, SKCM, and UCEC. TPM values were transformed into a log2(TPM+1) scale. (D) The results from the CPTAC dataset confirmed that PRKDC total protein is highly expressed in breast cancer, ovarian cancer, colon cancer, UCEC, and LUAD compared with normal controls, but not clear cell RCC. DLBC = lymphoid neoplasm diffuse large B-cell lymphoma, LGG = brain lower grade glioma, LUAD = lung adenocarcinoma, OV = ovarian serous cystadenocarcinoma, PRKDC = protein kinase, DNA-activated, catalytic subunit, RCC = renal cell carcinoma, SKCM = skin cutaneous melanoma, TIMER2.0 = Tumor Immune Estimation Resource, version 2, THYM = thymoma, UCEC = uterine corpus endometrial carcinoma, UCS = uterine carcinosarcoma. *P < .05; **P < .01; ***P < .001.

We adopted the TCGA and GTEx datasets to look at PRKDC expression levels across diverse tumors. The expression of PRKDC in the tumor tissues of cervical squamous cell carcinoma and endocervical adenocarcinoma (CESC), brain lower grade glioma (LGG), liver hepatocellular carcinoma (LIHC), LUAD, lung squamous cell carcinoma (LUSC), bladder urothelial carcinoma (BLCA), breast invasive carcinoma (BRCA), cholangiocarcinoma, lymphoid neoplasm diffuse large B-cell lymphoma, rectumadenocarcinoma, stomach adenocarcinoma (STAD), thymoma (THYM), uterine carcinosarcoma, colon adenocarcinoma (COAD), esophageal carcinoma (ESCA), and head and neck squamous cell carcinoma (HNSC) were higher compare with those in neighboring normal tissues, as illustrated in Fig. 1A and B. By contrast, PRKDC expression levels were significantly lower in tumor tissues of kidney renal clear cell carcinoma (KIRC), kidney renal papillary cell carcinoma (KIRP), and thyroid carcinoma (THCA). But, there was no significant difference in other tumors, including glioblastoma multiforme (GBM), kidney chromophobe (KICH), pancreatic adenocarcinoma (PAAD), pheochromocytoma and paraganglioma, prostate adenocarcinoma, UCEC, adrenocortical carcinoma (ACC), acute myeloid leukemia, ovarian serous cystadenocarcinoma (OV), sarcoma (SARC), skin cutaneous melanoma (SKCM), and testicular germ cell tumors (all *P* > .05), as indicated in Figure 1A and Figure S1A, Supplemental Digital Content, http://links.lww.com/MD/G879.

We applied the “Pathological Stage Plot” modular of GEPIA 2 to confirmed a positive association between PRKDC expression and advanced cancer stage in OV, SKCM, and UCEC (all *P* < .05, Fig. 1C) but not the others (Fig. S1B, Supplemental Digital Content, http://links.lww.com/MD/G879). The results from the CPTAC dataset confirmed that PRKDC total protein is highly expressed in breast cancer, ovarian cancer, colon cancer, UCEC, and LUAD compared with normal controls, but not clear cell RCC (Fig. 1D).

### 3.2. Survival analysis results

**Figure 2.**
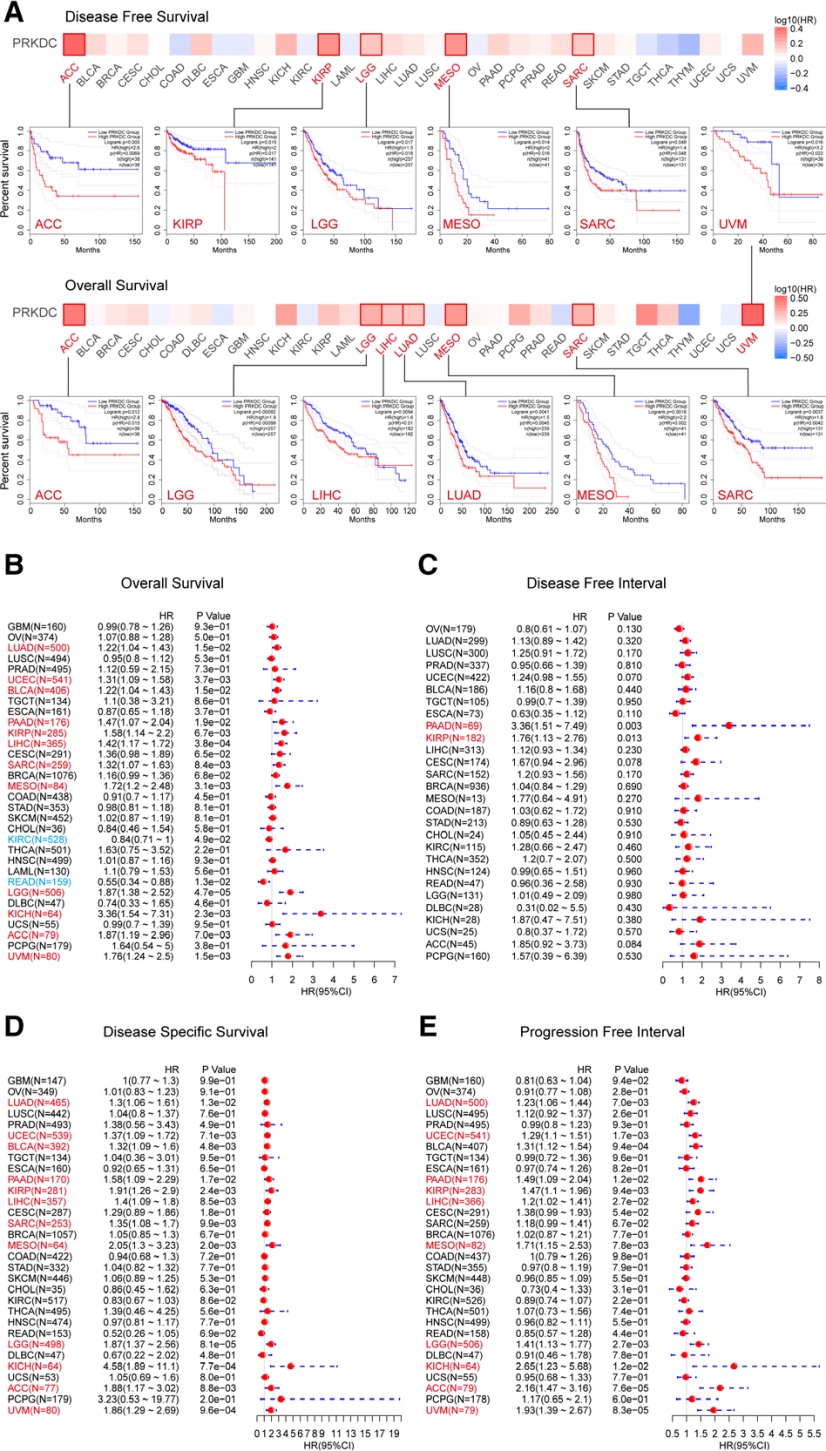
Prognosis value of PRKDC in pan-cancer base on different platforms. (A) GEPIA 2 platform was adopted to carry out overall survival and disease-free survival analysis in all TCGA tumors. A 50% cutoff value was chosen to separating the high or low PRKDC expression groups. Survival maps and Kaplan–Meier plots with significant results were presented at the same time. Forest plots of overall survival (B), disease-free interval (C), disease-special survival (D), and progression-free interval (E) comparing high and low PRKDC expression cohorts were performed by SangerBox online website. *P* < .05 is defined as significant. GEPIA 2 = gene expression profiling interactive analysis, version 2, PRKDC = protein kinase, DNA-activated, catalytic subunit, TCGA = The Cancer Genome Atlas.

We explored the prognosis value of PRKDC in pan-cancer base on several databases and platform. First, we evaluated the association between PRKDC and prognosis (OS and DFS) using GEPIA 2. As shown in Figures 2A, it suggested a poorer OS in high PRKDC expression cohort rather than low PRKDC expression cohort of LIHC (HR = 1.6, *P* = .01), ACC (HR = 2.8, *P* = .015), LGG (HR = 1.9, *P* = .00099), LUAD (HR = 1.5, *P* = .0045), mesothelioma (MESO) (HR = 2.2, *P* = .002), SARC (HR = 1.8, *P* = .0042), and UVM (HR = 3.2, *P* = .023). High PRKDC expression cohorts also exhibited shorter DFS than low PRKDC expression cohorts for ACC (HR = 2.6, *P* = .0069), KIRP (HR = 2, *P* = .017), LGG (HR = 1.5, *P* = .018), MESO (HR = 2, *P* = .016), and SARC (HR = 1.4, *P* = .048).

Next, survival data from the SangerBox platform revealed a link between high PRKDC expression and bad OS (Fig. 2B) for LUAD (*P* = .015), UCEC (*P* = .0037), BLCA (*P* = .015), PAAD (*P* = .019), KIRP (*P* = .0067), LIHC (*P* = .00038), SARC (*P* = .0084), MESO (*P* = .0031), LGG (*P* = .000047), KICH (*P* = .0023), ACC (*P* = .007) and UVM (*P* = .0015), but a greater OS in KIRC (*P* = .049) and rectumadenocarcinoma (*P* = .013). High PRKDC expression cohort was related to a shorter DFI (Fig. 2C) in PAAD (*P* = .003) and KIRP (*P* = .013) when compared with a low PRKDC expression cohort. Similar to OS, high PRKDC expression group showed worse DSS (Fig. 2D) compare with the low PRKDC expression group for LUAD (*P* = .013), UCEC (*P* = .0071), BLCA (*P* = .0048), PAAD (*P* = .017), KIRP (*P* = .0024), LIHC (*P* = .0085), SARC (*P* = .0099), MESO (*P* = .002), LGG (*P* = .000081), KICH (*P* = .00077), ACC (*P* = .0088), and UVM (*P* = .00096). Additionally, high PRKDC expression was relevant with shorter PFI for LUAD (*P* = .007), UCEC (*P* = .0017), PAAD (*P* = .012), KIRP (*P* = .0094), LIHC (*P* = .027), MESO (*P* = .0078), LGG (*P* = .0027), KICH (*P* = .012), ACC (*P* = .000076), and UVM (*P* = .000083).

Finally, we evaluated prognostic value of PRKDC in pan-cancer (OS and RFS) on Kaplan–Meier plotter database (https://kmplot.com/analysis/). The PRKDC level negatively correlated with the OS (Fig. S2, Supplemental Digital Content, http://links.lww.com/MD/G879) in following cancers: BLCA (*P* = .03), BRCA (*P* = .025), CESC (*P* = .003), KIRP (*P* = .0095), LIHC (*P* = .0067), LUAD (*P* = .001), PAAD (*P* = .0069), SARC (*P* = .00015), THCA (*P* = .0069), and UCEC (*P* = .0001), but was positively correlated with the OS in ESCA (*P* = .0058), KIRC (*P* = .0012), and THYM (*P* = .021). There was a negative relation between the PRKDC level and relapse-free survival (Fig. S3, Supplemental Digital Content, http://links.lww.com/MD/G879) in KIRP (*P* = .013), LIHC (*P* = .029), PAAD (*P* = .00038), THCA (*P* = .015), UCEC (*P* = .0061), and SARC (*P* = .011), but a positive correlation was shown in ESCA (*P* = .0075) and OV (*P* = .011).

The above data revealed that PRKDC is elevated in most human tumors, suggesting that it may serve as a biomarker for poor prognosis, though varied across different types of tumors.

### 3.3. PRKDC alteration analysis results

**Figure 3.**
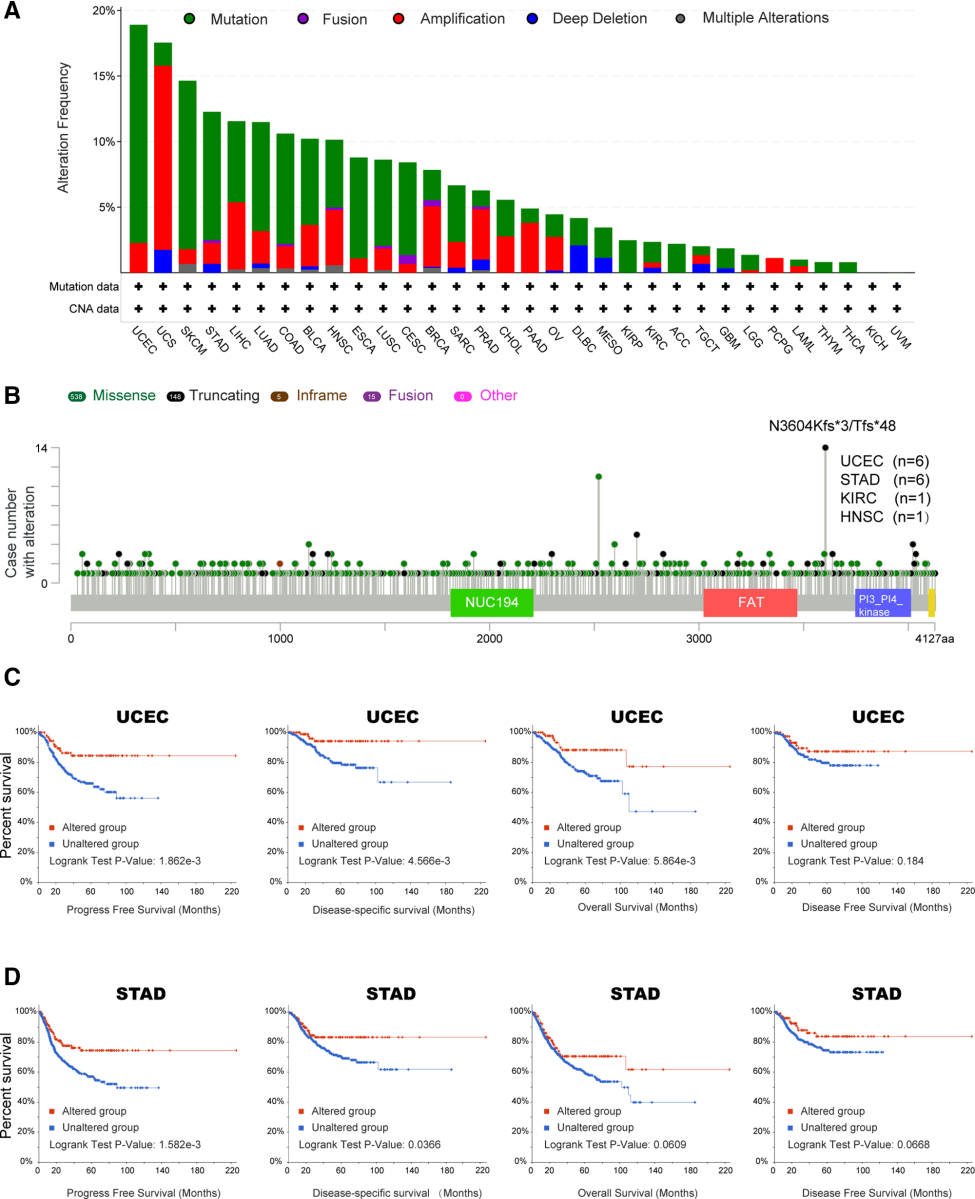
PRKDC mutation status in different tumor patients. Summary of PRKDC mutations in tumors was obtained from the cBioPortal tool, “mutation” showed the highest frequency of PRKDC alteration in most tumors (A) and the alteration types, mutated sites, and mutation cases were exhibited in a schematic representation of PRKDC protein structure (B). We also studied the correlation between mutation site (N3604Kfs*3/Tfs*48) and progression-free survival, disease-specific survival, overall survival, and disease-free survival of UCEC (C) and STAD (D). *P* < .05 is defined as significant. PRKDC = protein kinase, DNA-activated, catalytic subunit, STAD = stomach adenocarcinoma, UCEC = uterine corpus endometrial carcinoma.

Because genomic mutations are linked to tumor oncogenesis and progression, we observed 10,953 patients from 32 TCGA studies in total (https://www.cbioportal.org), and found that 804 (7%) of them carried at least one PRKDC gene mutation. As it is indicated in Figure 3A, “mutation” showed the highest frequency of PRKDC alteration in most tumors, including UCEC, SKCM, STAD, LIHC, LUAD, COAD, BLCA, HNSC, ESCA, LUSC, CESC, SARC, MESO, KIRP, KIRC, ACC, GBM, LGG, THYM, and THCA. In particular, “mutation” was the only genetic alteration type in KIRP (~2.5% frequency), ACC (~2% frequency), THYM (~1% frequency), and THCA (~1% frequency). PRKDC mutation frequency was the highest in UCEC (>18%), and the “amplification” alteration type was the primary type in uterine carcinosarcoma, with an alteration frequency nearby 15%.

The alteration types, mutated sites, and mutation cases numbers were exhibited in a schematic representation of PRKDC protein structure in Figure 3B. It showed that “Truncating” is the most common sort of genetic alteration, and N3604Kfs*3/Tfs*48 alteration was detected in 6 cases of UCEC and 6 cases of STAD.

Additionally, we compared the survival prognosis in cohorts with or without PRKDC genetic alteration across different tumors. It indicated a better prognosis for PFS (*P* = 1.862e-03), DSS (*P* = 4.566e-03), and OS (*P* = 5.864e-03) in UCEC cases (Fig. 3C) with altered PRKDC, and better prognosis for PFS (*P* = 1.582e-03) and DSS (*P* = .0366) in STAD cases (Fig. 3D) with altered PRKDC, compared with cases without PRKDC alteration.

### 3.4. Protein phosphorylation analysis results

**Figure 4.**
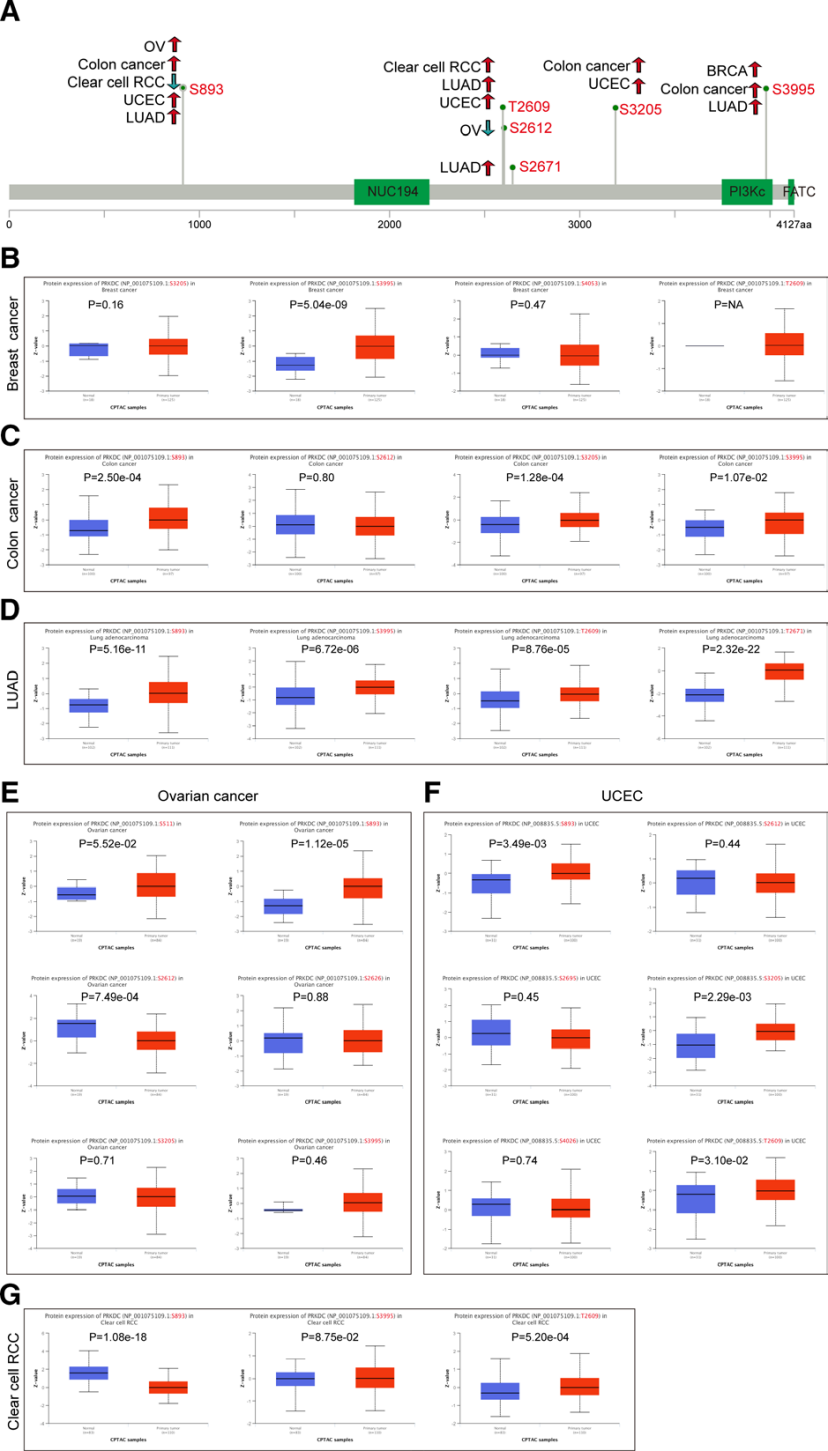
Phosphorylation analysis of PRKDC protein across tumors. (A) The schematic diagram summarizes the PRKDC phosphorylation sites and related tumors. We compared the PRKDC phosphoprotein expression level between normal tissue and tumor tissue of breast cancer (B), colon cancer (C), LUAD (D), ovarian cancer (E), UCEC (F), and clear cell RCC (G) via the UALCAN. *P* < .05 is defined as significant. LUAD = lung adenocarcinoma, PRKDC = protein kinase, DNA-activated, catalytic subunit, RCC = renal cell carcinoma, UCEC = uterine corpus endometrial carcinoma.

Using the CPTAC dataset, we evaluated the PRKDC phosphorylation level in tumor tissues as well normal tissues in 6 kinds of cancers, including breast cancer, colon cancer, clear cell RCC, UCEC, LUAD, and ovarian cancer. A schematic diagram summarizes the PRKDC phosphorylation sites and related tumors (Fig. 4A). The S893 locus of PRKDC exhibited an increased phosphorylation levels in colon cancer (Fig. 4C, *P* = 2.5e-04), LUAD (Fig. 4D, *P* = 5.16e-11), ovarian cancer (Fig. 4E, *P* = 1.12e-05), and UCEC (Fig. 4F, *P* = 3.49 e-03), but a decreased phosphorylation level in clear cell RCC (Fig. 4G, *P* = 1.08e-18). Additionally, we confirmed that PRKDC phosphorylation of S893 was experimentally proved by one research^[25]^ through the phosphoNET database (http://www.phosphonet.ca/). To learn more about the significance of S893 phosphorylation in carcinogenesis, further molecular investigations are needed.

### 3.5. Immune infiltration analysis results

**Figure 5.**
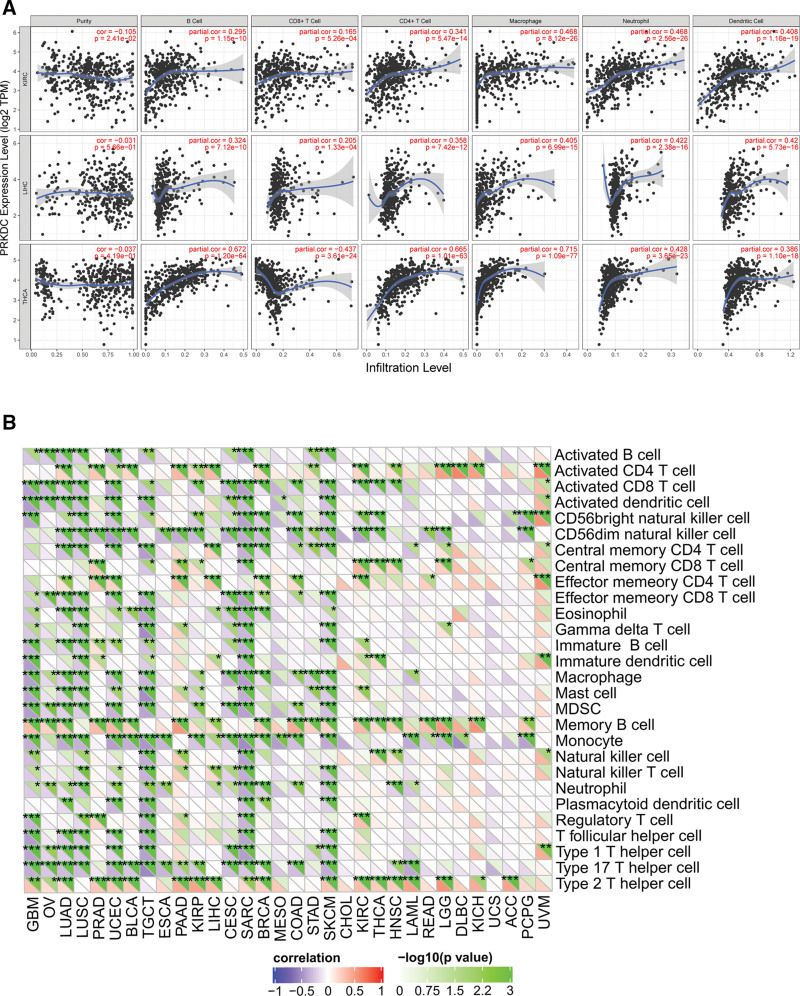
Coefficient of TIICs and PRKDC across TCGA tumors. (A) KIRC, LIHC, and THCA show the strongest association of PRKDC expression and immune infiltrating. (B) We identified and evaluated the relationship between PRKDC expression and 28 immune cell subtypes via SangerBox online tool. *P < .05, **P < .01, and ***P < .001. KIRC = kidney renal clear cell carcinoma, LIHC = liver hepatocellular carcinoma, PRKDC = protein kinase, DNA-activated, catalytic subunit, TCGA = The Cancer Genome Atlas, THCA = thyroid carcinoma, TIICs = tumor-infiltrating immune cells.

TIICs from tumor microenvironment (TME) are correlated with initiation, progression or metastasis of tumor,^[26]^ so we explored the coefficient of TIICs and PRKDC expression in diverse tumors of TCGA by using TIMER database. According to the findings, PRKDC was relevant to tumor purity in 9 different kinds of cancers, and also remarkably associated with the infiltration levels of B cells, CD8+ T cells, CD4+ T cells, macrophages, neutrophils, and DCs in 15, 21, 14, 18, 21, and 19 kinds of tumors, separately (Fig. 5A and Fig S4–7, Supplemental Digital Content, http://links.lww.com/MD/G879). In addition, KIRC, LIHC, and THCA show the most strongly association between PRKDC and immune infiltrating levels (Fig. 5B). For KIRC, a negative interrelation was confirmed between PRKDC gene and tumor purity (*R* = −0.105, *P* = 2.41e-2), but PRKDC expression and B cells (*R* = 0.295, *P* = 1.15e-10), CD8+ T cells (*R* = 0.165, *P* = 5.26e-04), CD4+ T cells (*R* = 0.341, *P* = 5.47e-14), macrophages (*R* = 0.468, *P* =8.12e-26), neutrophils (*R* = 0.468, *P* = 2.56e-26), and DCs (*R* = 0.408, *P* = 1.16e-19) indicated a positive association. In LIHC, PRKDC expression was positively correlated with B cells (*R* = 0.324, *P* = 7.12e-10), CD8+ T cells (*R* = 0.205, *P* = 1.33e-04), CD4+ T cells (*R* = 0.358, *P* = 7.42e-12), macrophages (*R* = 0.405, *P* = 6.99e-15), neutrophils (*R* = 0.422, *P* =2.38e-16), and DCs (*R* = 0.42, *P* =5.73e-16), but was not associated with tumor purity. About THCA, PRKDC gene shown a negative association with CD8+ T cells (*R* = −0.437, *P* = 3.61e-24), but there was a positive association with B cells (*R* = 0.672, *P* = 1.20e-64), CD4+ T cells (*R* = 0.665, *P* = 1.01e-63), macrophages (*R* = 0.715, *P* = 1.09e-77), neutrophils (*R* = 0.428, *P* = 3.65e-23), and DCs (*R* = 0.386, *P* = 1.10e-18), but was not related to tumor purity. We employed the SangerBox online tool to discuss the relevance between PRKDC and immune cell subtypes in the TME (Fig. 5B). The results indicated that CD56dim natural killer cell, memory B cell, monocyte and type 2 T helper cell had the strongest correlation with PRKDC expression.

### 3.6. Immune checkpoint genes, TMB, and MSI analysis results

**Figure 6.**
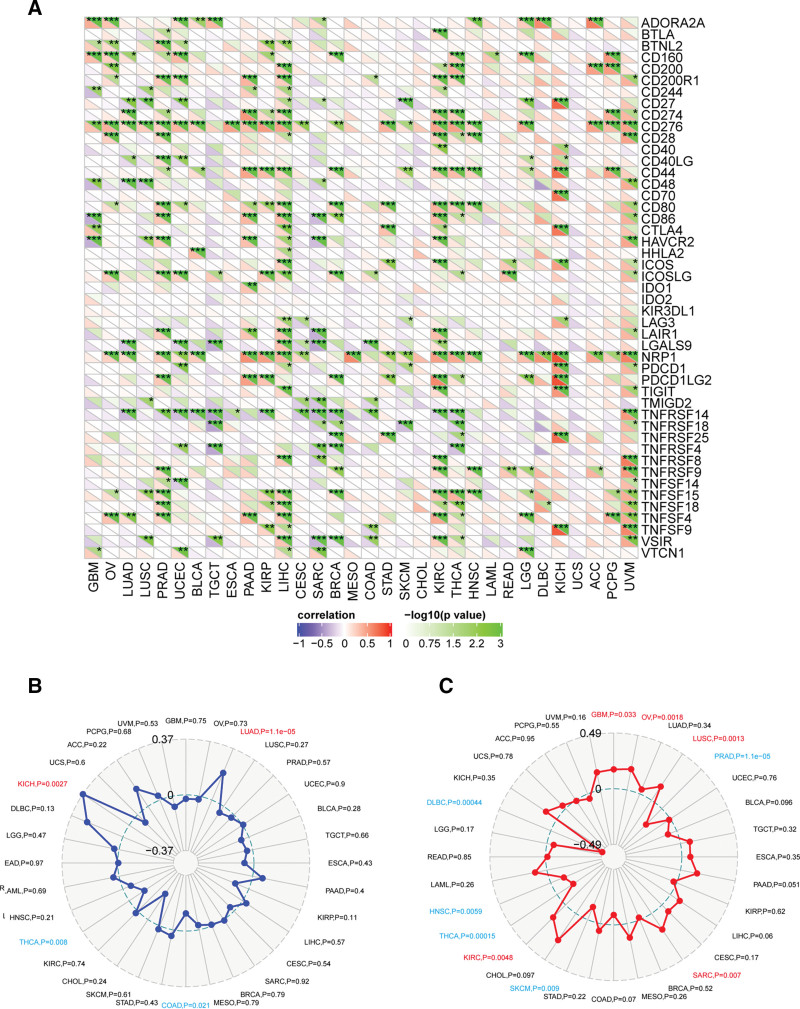
Correlation of PRKDC expression and checkpoint genes, TMB and MSI in tumors. (A) Relationship between PRKDC and 47 distinct immune checkpoint genes across different tumors. (B) The radar plot displays the association of the PRKDC gene and TMB. (C) The radar plot displays the association of the PRKDC gene and MSI. *P < .05, **P < .01, and ***P < .001. MSI = microsatellite instability, PRKDC = protein kinase, DNA-activated, catalytic subunit, TMB = tumor mutation burden.

Immune checkpoints are important immune system regulator. Some tumors can protect themselves from attack by taking advantage of immune checkpoint genes. We used SangerBox platform to investigate the association between PRKDC and the immune checkpoint genes across different tumors, as shown in Figure 6A. For example, in LIHC, PRKDC was positively correlated with expression of CD200, CD200R1, CD244, CD27, BTNL2, CD274, CD276, CD44, HAVCR4, CD28, CD80, CD86, CTLA4, HHLA2, ICOSLG, LGALS9, NRP1, TNFSF15, ICOS, LAG3, LAIR1, PDCD1, TNFSF4, TNFSF9, VSIR, VTCN1, TIGIT, TNFRSF8, and TNFSF18. TMB and MSI are important factors that influence tumor initiation and development, as well as tumor immunotherapy response. Higher TMB was related to better OS and better outcomes from immune checkpoint inhibitor (ICI) therapy in tumor patients.^[27]^ Radar plots (Fig. 6B) showed a positive association between PRKDC and TMB in KICH (*P* = .0027) and LUAD (*P* = 1.1e-05), but a negative association in THCA (*P* = .008) and COAD (*P* = .021). We also observed a positive association between PRKDC expression and MSI for GBM (*P* = .033), OV (*P* = .0018), LUSC (*P* = .0013), SARC (*P* = .007), and KIRC (*P* = .0048) but a negative correlation for prostate adenocarcinoma (*P* = 1.1e-05), SKCM (*P* = .009), THCA (*P* = .00015), HNSC (*P* = .0059), and lymphoid neoplasm diffuse large B-cell lymphoma (*P* = .00044) (Fig. 6C). In summary, all these data suggested a broad association between high PRKDC expression and tumor immunity.

### 3.7. PPI network and enrichment analysis results

**Figure 7.**
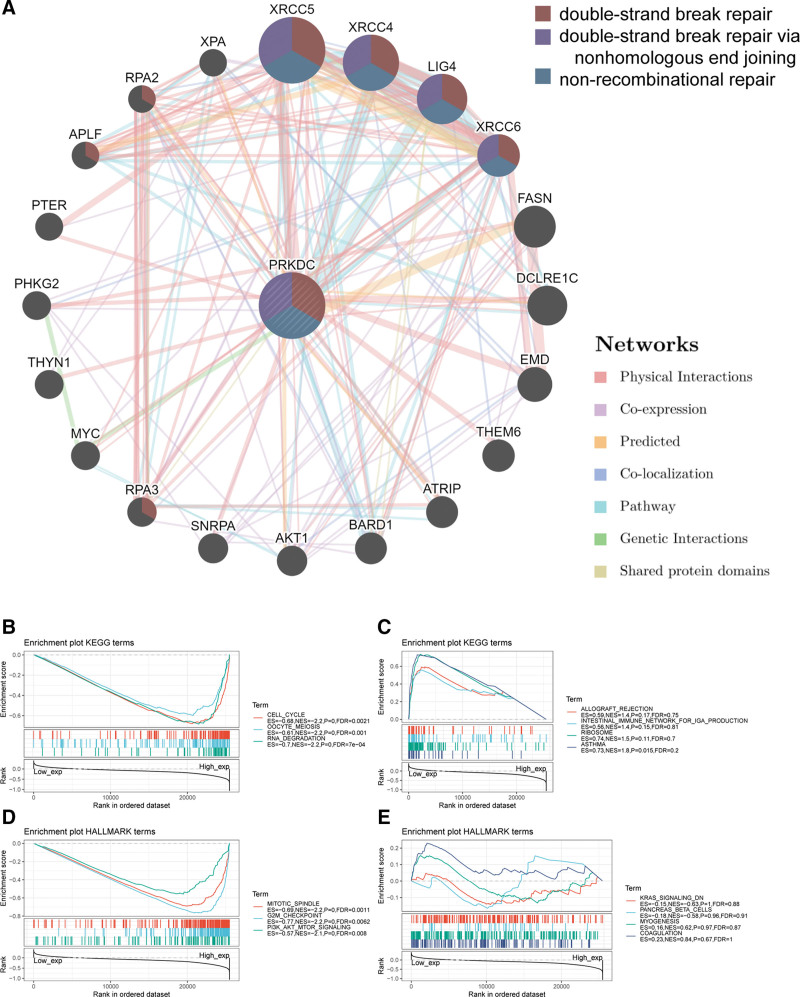
PRKDC-related genes enrichment analysis. (A) GeneMANIA online platform built a PPI network for PRKDC. Enriched gene sets by KEGG analysis in samples with high PRKDC expression (B) and low PRKDC expression (C). Enriched gene sets by HALLMARK analysis in samples with high PRKDC expression (D) and low PRKDC expression (E). Gene sets with |NES|>1, NOM *P* < .01, and FDR q < 0.25 were defined as significant. The plot only showed a few of the most significant gene sets. KEGG = Kyoto encyclopedia of genes and genomes, PRKDC = protein kinase, DNA-activated, catalytic subunit, PPI = protein–protein interaction.

To investigated the mechanism of PRKDC in tumorigenesis, this work employed GeneMANIA online platform to build a PPI network for PRKDC. As it is shown in Figure 7A, PRKDC shared a same pathway with XRCC4, XRCC5, XRCC6, and LIG4, and PRKDC had strong physical interactions with XRCC5 and XRCC6. The aforementioned 2 genes encode Ku80 and Ku70, respectively, which make up a heterodimer protein critical for NHEJ pathway of DNA repair. As a regulatory subunit, the Ku80/Ku70 heterodimer can induce a 100-fold increase in affinity between DNA-PKcs and DNA. LIG4 is also required for NHEJ because it can form a complex with XRCC4 that further interacts with the DNA-PK.^[28]^

Enrichment analysis of high PRKDC expression and low PRKDC expression was performed using GSEA (Fig. 7B–E). The top few KEGG enrichment terms in the high PRKDC expression group named “cell cycle,” “oocyte meiosis,” and “RNA-degradation.” HALLMARK revealed enrichment terms of high PRKDC expression in “mitotic spindle,” “G2M check point,” and “P13K-AKT-MTOR signaling.”

## 4. Discussion

Functional associations between PRKDC and various tumors have been reported in many publications.^[10,29–33]^ It is unclear that whether PRKDC is involved in the etiology of various cancers via common molecular processes. There was no publication with PRKDC pan-cancer analysis from a holistic oncology approach, according to a literature search. Therefore, we conducted a comprehensive study of PRKDC genes in 33 types of cancers based on TCGA, GTEx, and CPTAC databases, including gene expression, gene alteration, protein phosphorylation, TME, and biological pathways.

Inducing irreversible DNA damage is considered to be a significant therapeutic strategy for cancer. DNA-dependent protein kinase complex (DNA-PK) consisted of DNA-PKcs and Ku80/Ku70 heterodimer protein, and it is very important to DSB repair response. Thus, it explains why DNA-PK is associated with a reduced response to DNA-damaging drugs as well as treatment resistance in various malignancies.^[7,34,35]^ This study confirmed that PRKDC was substantially expressed in most tumors and was linked to a poor prognosis in a lot of tumor types.^[36]^ The development of PRKDC targeted therapy has been prompted by these evidences and the fact that DNA-PKcs is a latent treatment target.^[37,38]^ There are many pathways that can promote tumor cells survival and proliferation, so knowing PRKDC’s regulatory mechanisms is crucial for designing effective therapies to inhibit PRKDC.

Only 10 cases have been recorded with disease-causing mutations in PRKDC. Six of them exhibited severe combined immunodeficiency, including granulomas and autoimmunity.^[39–41]^ It results from that PRKDC mutation decreases affinity between DNA-PKcs and DNA and jeopardizes the activity of Artemis, which is necessary for V(D)J recombination as well. We found that over 18% of UCEC patients and about 12% of STAD patients presented alteration in PRKDC and the alteration correlates with better survival. In a recent study,^[42]^ PRKDC mutation was found to be related to higher TMB, elevated mRNA levels of immunity-related genes as well as improved response to ICI treatments in tumor individual. Another publication also discovered that PRKDC mutation was linked to an advanced TMB and MSI states in tumors, and knocked down PRKDC or used a DNA-PKcs inhibitor might improve ICI efficacy.^[43]^ Above findings demonstrate that the PRKDC mutation might be used as a biomarker in immunotherapy.

We used the CPTAC dataset to compared the DNA-PKcs phosphorylation levels in the following 6 types of tumors: LUAD, colon cancer, clear cell RCC, breast cancer, UCEC, and ovarian cancer. It indicated a higher phosphorylation level at the S893, T2609, S2671, S3205, and S3995 locus in the primary tumors than normal controls. In vitro, DNA-PKcs experiences substantial autophosphorylation, causing it to dissociate from Ku-bound DNA and lose its kinase function.^[44]^ In contrast, the capacity of DNA-PKcs to disassociate from Ku-DNA was diminished when the T2609 phosphorylation site was changed to alanine.^[45,46]^ As a result, DNA-PKcs autophosphorylation was considered critical for the DNA repair process.

PRKDC expression has been linked to various levels of immune infiltration in tumors, particularly in KIRC, LIHC, and THCA, according to this study. There was a strong negative relevance between PRKDC and tumor purity in KIRC, showing that PRKDC is relatively abundant in the TME. However, the degree of PRKDC expression in LIHC and THCA was independent of tumor purity, indicating that it was expressed equally among tumor cell and the TME. For all 3 tumors, it showed a clearly positive associations between PRKDC expression and TIICs, but a negative association with CD8+ T cells in THCA. These distinctions indicated that there were variances among tumors in TME.

In the TME, immune system could identify and kill tumor cells. However, tumor cells can use a variety of tactics to survival and proliferate that inhibit the immune system.^[47,48]^ Tumor immunotherapy, which includes of therapeutic antibodies, ICIs, tumor vaccines, and cell treatments, can help the body regain its natural antitumor immune response. We looked examined the link between PRKDC expression and 47 different immunological checkpoint genes, and found that high PRKDC expression may play a key role in immune evasion.

MSI was considered a biomarker for identifying individuals who may benefit from immunotherapies since it is linked to a higher cancer risk with important clinicopathological features, such as elevated TMB and more TIICs.^[49]^ TMB might be adopted as a biomarker to forecast how well checkpoint blockades may work.^[50,51]^ We showed evidence of a link between PRKDC expression and MSI or TMB across pan-cancers in this work, as well as the potential function of PRKDC in oncology immunological and as a predictive biomarker for a variety of malignancies.

In conclusion, our study revealed statistical associations between PRKDC expression and clinical outcome, genetic mutation, protein phosphorylation, immune cells infiltration, immune checkpoints, TMB, and MSI across pan-cancers, and confirmed PRKDC’s latent involvement in tumor immunology and as a therapeutic target and prognostic biomarker. Unfortunately, this work merely performed a bioinformatics analysis of PRKDC across several databases and platforms, lacking of in vitro or in vivo experiments. Therefore, further cellular and molecular mechanistic research on PRKDC is desired to better understand the role of PRKDC in tumors.

### Author contributions

X.S. and X.Y. designed this work. F.Y. performed the bioinformatics analyses. All authors participated in writing the manuscript. All authors read and approved the final manuscript.

### Acknowledgments

The authors sincerely acknowledge the publicly available of the GTEx (https://gtexportal.org/home/) database and TCGA (https://www.cancer.gov/) database.

## Supplementary Material


