# Supplementary material for: Potential value of PRKDC as a therapeutic target and prognostic biomarker in pan-cancer
Source: Medicine (Baltimore). 2022 Jul 8;101(27):e29628. doi: 10.1097/MD.0000000000029628 (PMC9259106; doi:10.1097/MD.0000000000029628)
Supplement: Supplementary file 1 [file medi-101-e29628-s001.pdf]

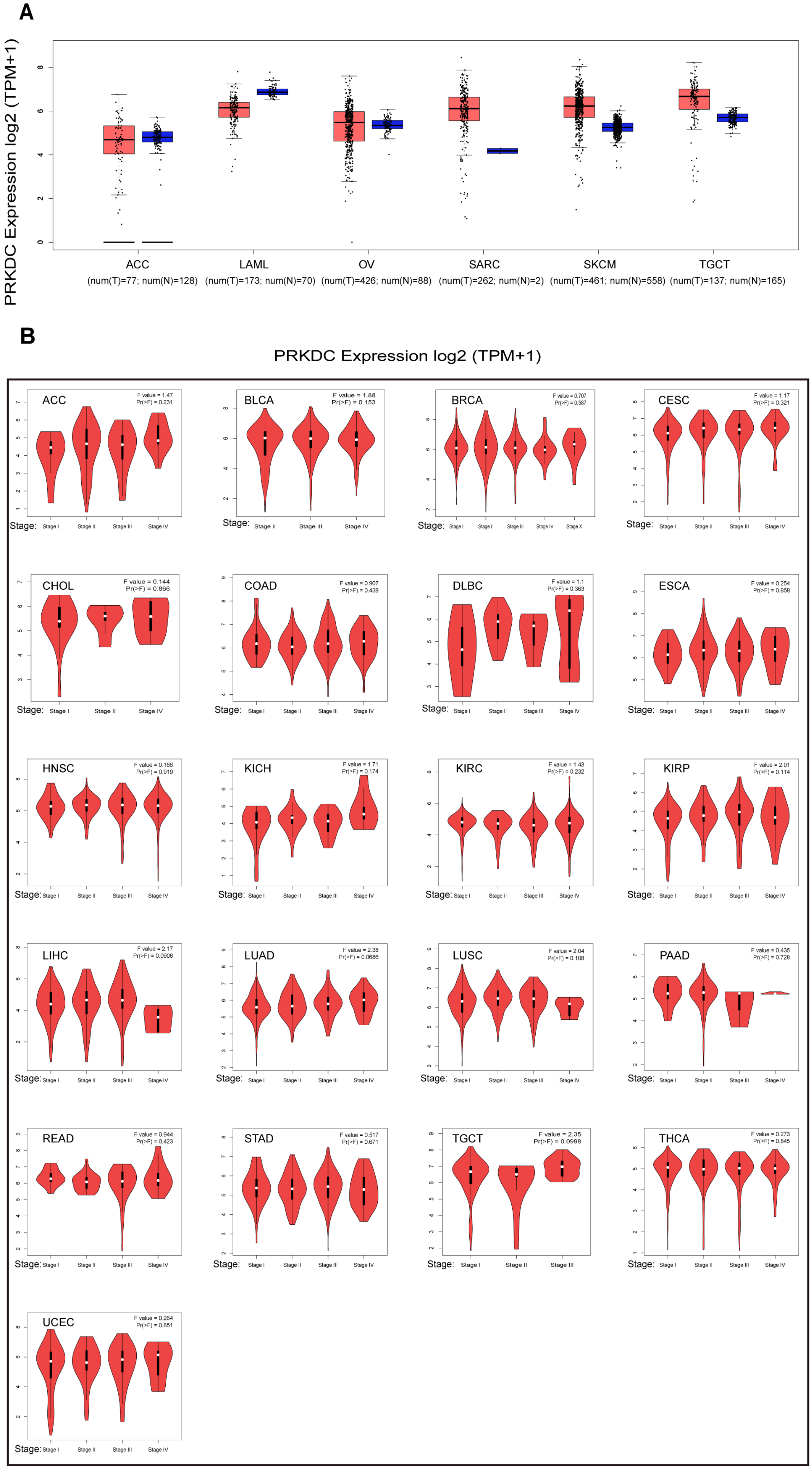

A
PRKDC Expression log2 (TPM+1)
ACC
(num(T)=77; num(N)=128)
LAML
(num(T)=173; num(N)=70)
OV
(num(T)=426; num(N)=88)
SARC
(num(T)=262; num(N)=2)
SKCM
(num(T)=461; num(N)=558)
TGCT
(num(T)=137; num(N)=165)
B
PRKDC Expression log2 (TPM+1)
ACC F value = 1.47 Pr(>F) = 0.231
BLCA F value = 1.88 Pr(>F) = 0.153
BRCA F value = 0.707 Pr(>F) = 0.587
CESC F value = 1.17 Pr(>F) = 0.321
CHOL F value = 0.144 Pr(>F) = 0.866
COAD F value = 0.907 Pr(>F) = 0.438
DLBC F value = 1.1 Pr(>F) = 0.363
ESCA F value = 0.254 Pr(>F) = 0.858
HNSC F value = 0.166 Pr(>F) = 0.919
KICH F value = 1.71 Pr(>F) = 0.174
KIRC F value = 1.43 Pr(>F) = 0.232
KIRP F value = 2.01 Pr(>F) = 0.114
LIHC F value = 2.17 Pr(>F) = 0.0908
LUAD F value = 2.38 Pr(>F) = 0.0686
LUSC F value = 2.04 Pr(>F) = 0.108
PAAD F value = 0.435 Pr(>F) = 0.728
READ F value = 0.944 Pr(>F) = 0.423
STAD F value = 0.517 Pr(>F) = 0.671
TGCT F value = 2.35 Pr(>F) = 0.0998
THCA F value = 0.273 Pr(>F) = 0.845
UCEC F value = 0.264 Pr(>F) = 0.851
Stage: Stage I Stage II Stage III Stage IV Stage X

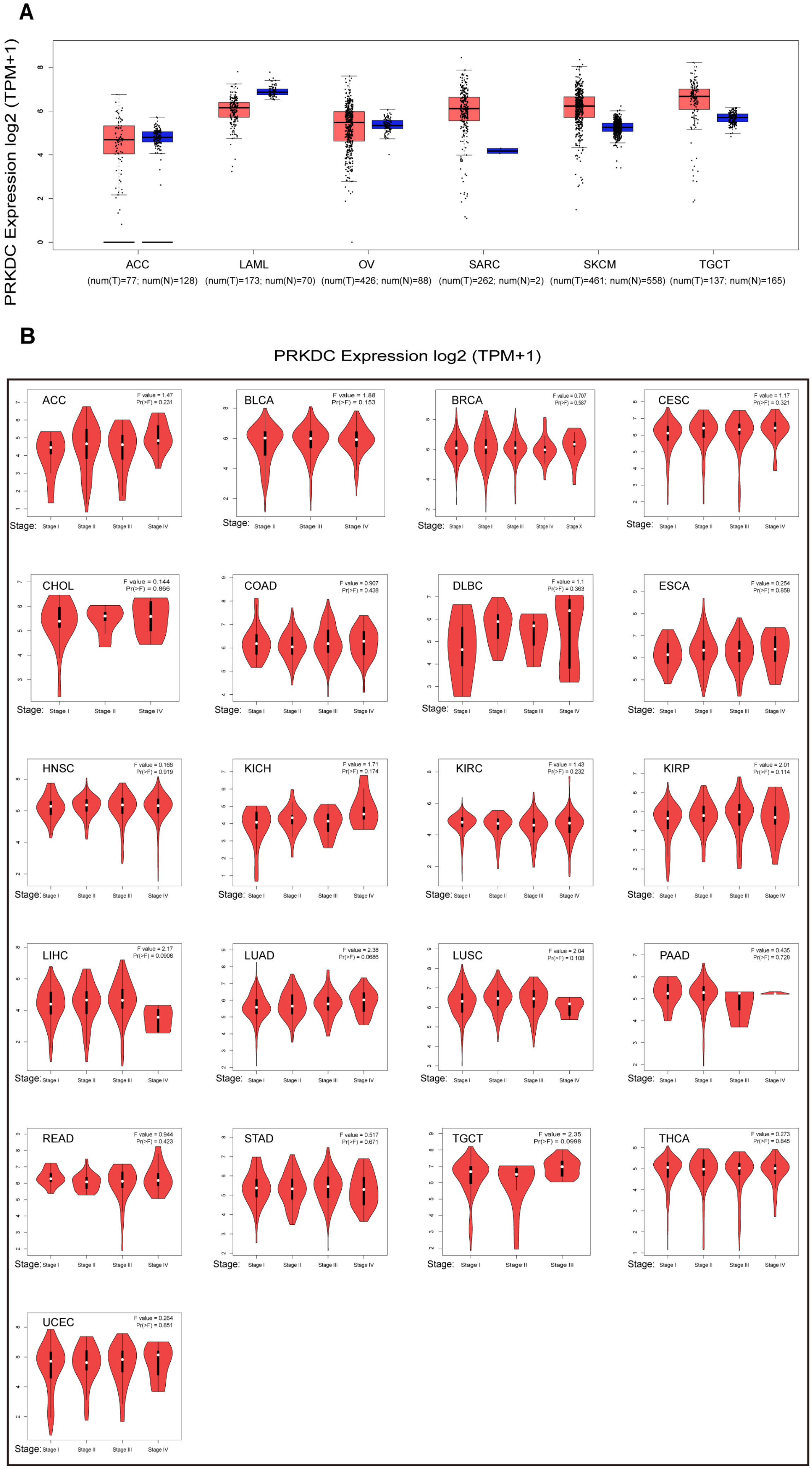
A
PRKDC Expression log2 (TPM+1)
ACC (num(T)=77; num(N)=128)
LAML (num(T)=173; num(N)=70)
OV (num(T)=426; num(N)=88)
SARC (num(T)=262; num(N)=2)
SKCM (num(T)=461; num(N)=558)
TGCT (num(T)=137; num(N)=165)
B
PRKDC Expression log2 (TPM+1)
ACC F value = 1.47 Pr(>F) = 0.231
BLCA F value = 1.88 Pr(>F) = 0.153
BRCA F value = 0.707 Pr(>F) = 0.587
CESC F value = 1.17 Pr(>F) = 0.321
CHOL F value = 0.144 Pr(>F) = 0.866
COAD F value = 0.907 Pr(>F) = 0.438
DLBC F value = 1.1 Pr(>F) = 0.363
ESCA F value = 0.254 Pr(>F) = 0.858
HNSC F value = 0.166 Pr(>F) = 0.919
KICH F value = 1.71 Pr(>F) = 0.174
KIRC F value = 1.43 Pr(>F) = 0.232
KIRP F value = 2.01 Pr(>F) = 0.114
LIHC F value = 2.17 Pr(>F) = 0.0908
LUAD F value = 2.38 Pr(>F) = 0.0686
LUSC F value = 2.04 Pr(>F) = 0.108
PAAD F value = 0.435 Pr(>F) = 0.728
READ F value = 0.944 Pr(>F) = 0.423
STAD F value = 0.517 Pr(>F) = 0.671
TGCT F value = 2.35 Pr(>F) = 0.0998
THCA F value = 0.273 Pr(>F) = 0.845
UCEC F value = 0.264 Pr(>F) = 0.851
Stage:

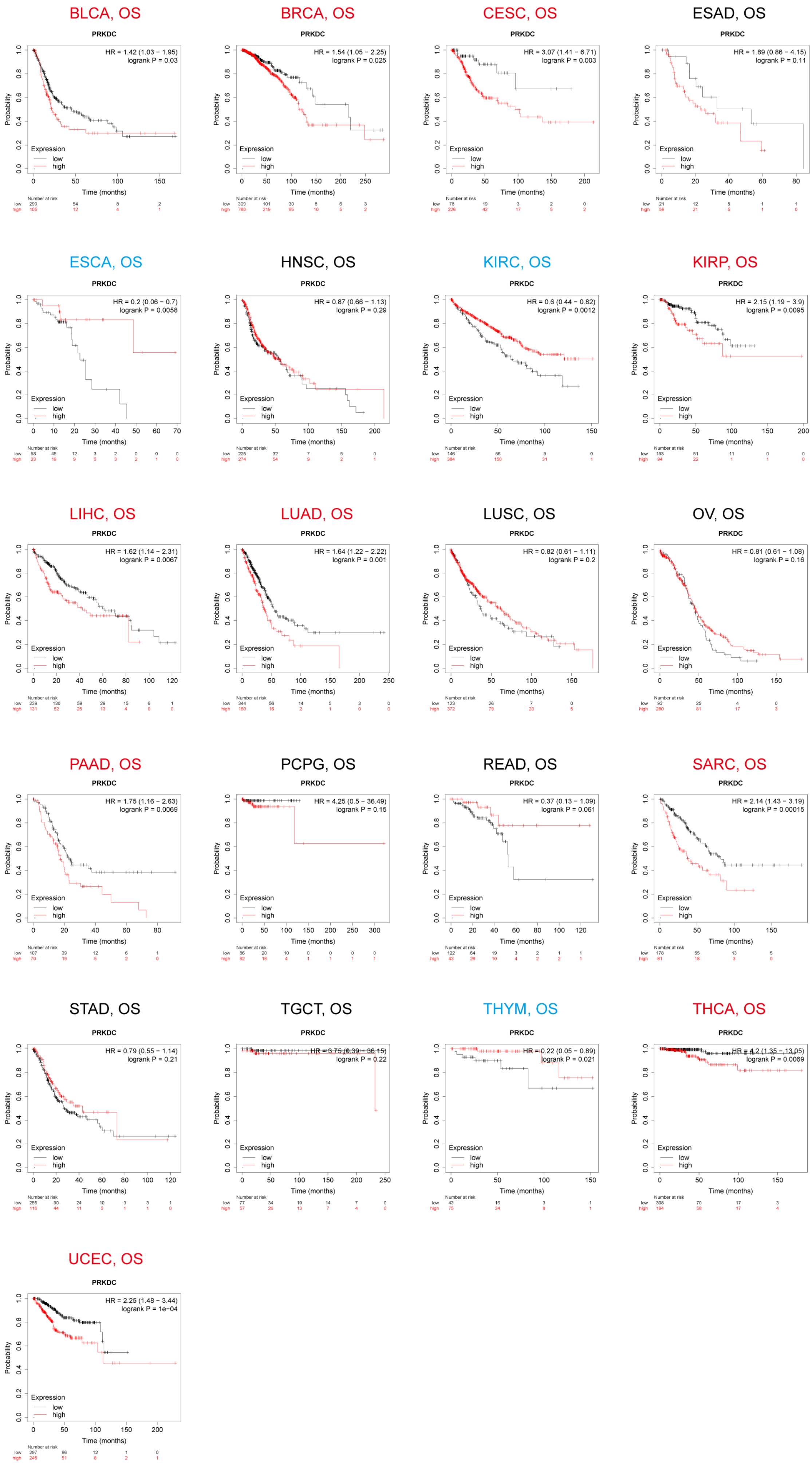

BLCA, OS
PRKDC
HR = 1.42 (1.03 − 1.95)
logrank P = 0.03
BRCA, OS
PRKDC
HR = 1.54 (1.05 − 2.25)
logrank P = 0.025
CESC, OS
PRKDC
HR = 3.07 (1.41 − 6.71)
logrank P = 0.003
ESAD, OS
PRKDC
HR = 1.89 (0.86 − 4.15)
logrank P = 0.11
ESCA, OS
PRKDC
HR = 0.2 (0.06 − 0.7)
logrank P = 0.0058
HNSC, OS
PRKDC
HR = 0.87 (0.66 − 1.13)
logrank P = 0.29
KIRC, OS
PRKDC
HR = 0.6 (0.44 − 0.82)
logrank P = 0.0012
KIRP, OS
PRKDC
HR = 2.15 (1.19 − 3.9)
logrank P = 0.0095
LIHC, OS
PRKDC
HR = 1.62 (1.14 − 2.31)
logrank P = 0.0067
LUAD, OS
PRKDC
HR = 1.64 (1.22 − 2.22)
logrank P = 0.001
LUSC, OS
PRKDC
HR = 0.82 (0.61 − 1.11)
logrank P = 0.2
OV, OS
PRKDC
HR = 0.81 (0.61 − 1.08)
logrank P = 0.16
PAAD, OS
PRKDC
HR = 1.75 (1.16 − 2.63)
logrank P = 0.0069
PCPG, OS
PRKDC
HR = 4.25 (0.5 − 36.49)
logrank P = 0.15
READ, OS
PRKDC
HR = 0.37 (0.13 − 1.09)
logrank P = 0.061
SARC, OS
PRKDC
HR = 2.14 (1.43 − 3.19)
logrank P = 0.00015
STAD, OS
PRKDC
HR = 0.79 (0.55 − 1.14)
logrank P = 0.21
TGCT, OS
PRKDC
HR = 3.75 (0.39 − 36.15)
logrank P = 0.22
THYM, OS
PRKDC
HR = 0.22 (0.05 − 0.89)
logrank P = 0.021
THCA, OS
PRKDC
HR = 4.2 (1.35 − 13.05)
logrank P = 0.0069
UCEC, OS
PRKDC
HR = 2.25 (1.48 − 3.44)
logrank P = 1e−04
Probability
Time (months)
Expression
low
high
Number at risk

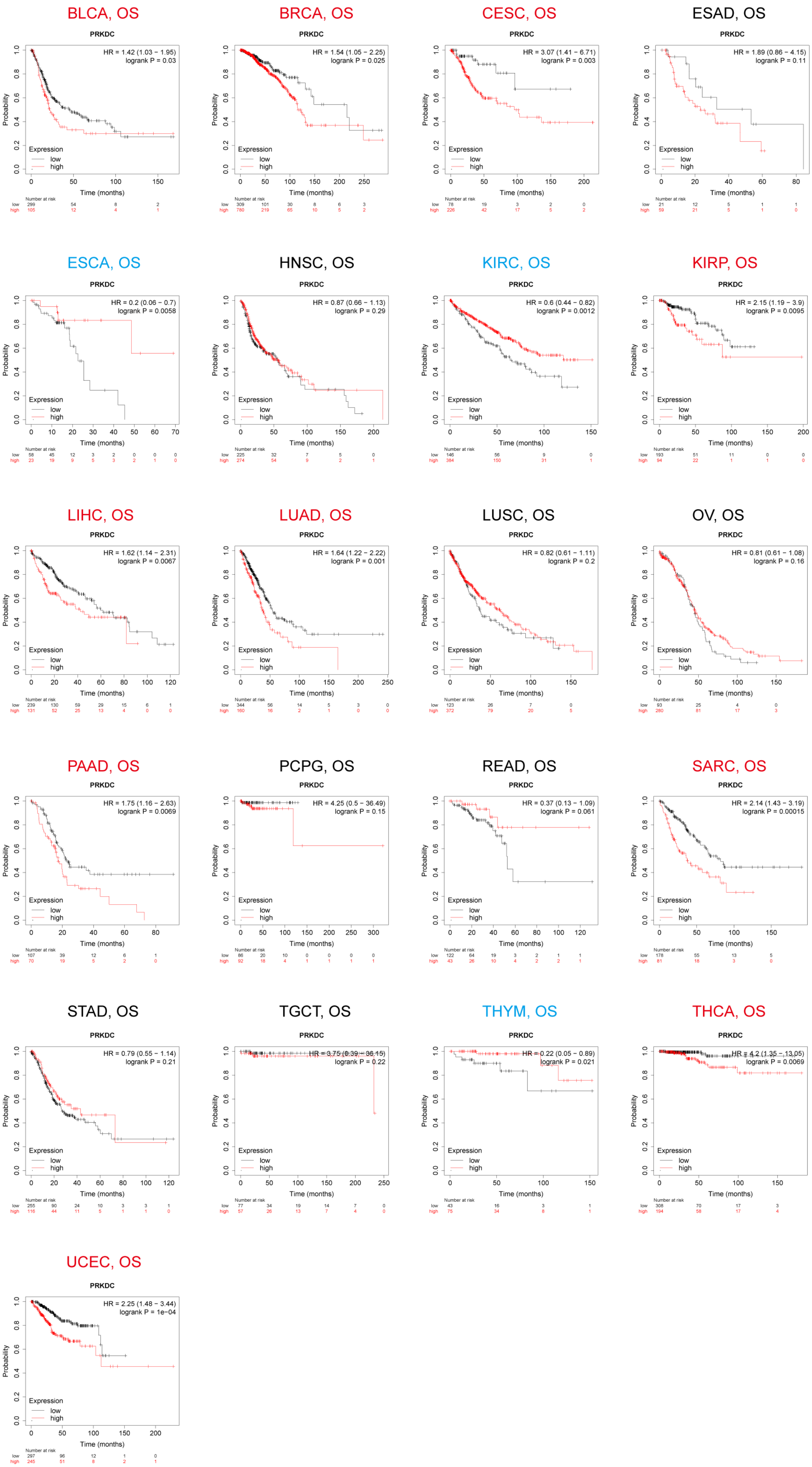
BLCA, OS
PRKDC
HR = 1.42 (1.03 − 1.95)
logrank P = 0.03
BRCA, OS
PRKDC
HR = 1.54 (1.05 − 2.25)
logrank P = 0.025
CESC, OS
PRKDC
HR = 3.07 (1.41 − 6.71)
logrank P = 0.003
ESAD, OS
PRKDC
HR = 1.89 (0.86 − 4.15)
logrank P = 0.11
ESCA, OS
PRKDC
HR = 0.2 (0.06 − 0.7)
logrank P = 0.0058
HNSC, OS
PRKDC
HR = 0.87 (0.66 − 1.13)
logrank P = 0.29
KIRC, OS
PRKDC
HR = 0.6 (0.44 − 0.82)
logrank P = 0.0012
KIRP, OS
PRKDC
HR = 2.15 (1.19 − 3.9)
logrank P = 0.0095
LIHC, OS
PRKDC
HR = 1.62 (1.14 − 2.31)
logrank P = 0.0067
LUAD, OS
PRKDC
HR = 1.64 (1.22 − 2.22)
logrank P = 0.001
LUSC, OS
PRKDC
HR = 0.82 (0.61 − 1.11)
logrank P = 0.2
OV, OS
PRKDC
HR = 0.81 (0.61 − 1.08)
logrank P = 0.16
PAAD, OS
PRKDC
HR = 1.75 (1.16 − 2.63)
logrank P = 0.0069
PCPG, OS
PRKDC
HR = 4.25 (0.5 − 36.49)
logrank P = 0.15
READ, OS
PRKDC
HR = 0.37 (0.13 − 1.09)
logrank P = 0.061
SARC, OS
PRKDC
HR = 2.14 (1.43 − 3.19)
logrank P = 0.00015
STAD, OS
PRKDC
HR = 0.79 (0.55 − 1.14)
logrank P = 0.21
TGCT, OS
PRKDC
HR = 3.75 (0.39 − 36.15)
logrank P = 0.22
THYM, OS
PRKDC
HR = 0.22 (0.05 − 0.89)
logrank P = 0.021
THCA, OS
PRKDC
HR = 4.2 (1.35 − 13.05)
logrank P = 0.0069
UCEC, OS
PRKDC
HR = 2.25 (1.48 − 3.44)
logrank P = 1e−04
Probability
Time (months)
Expression
low
high
Number at risk

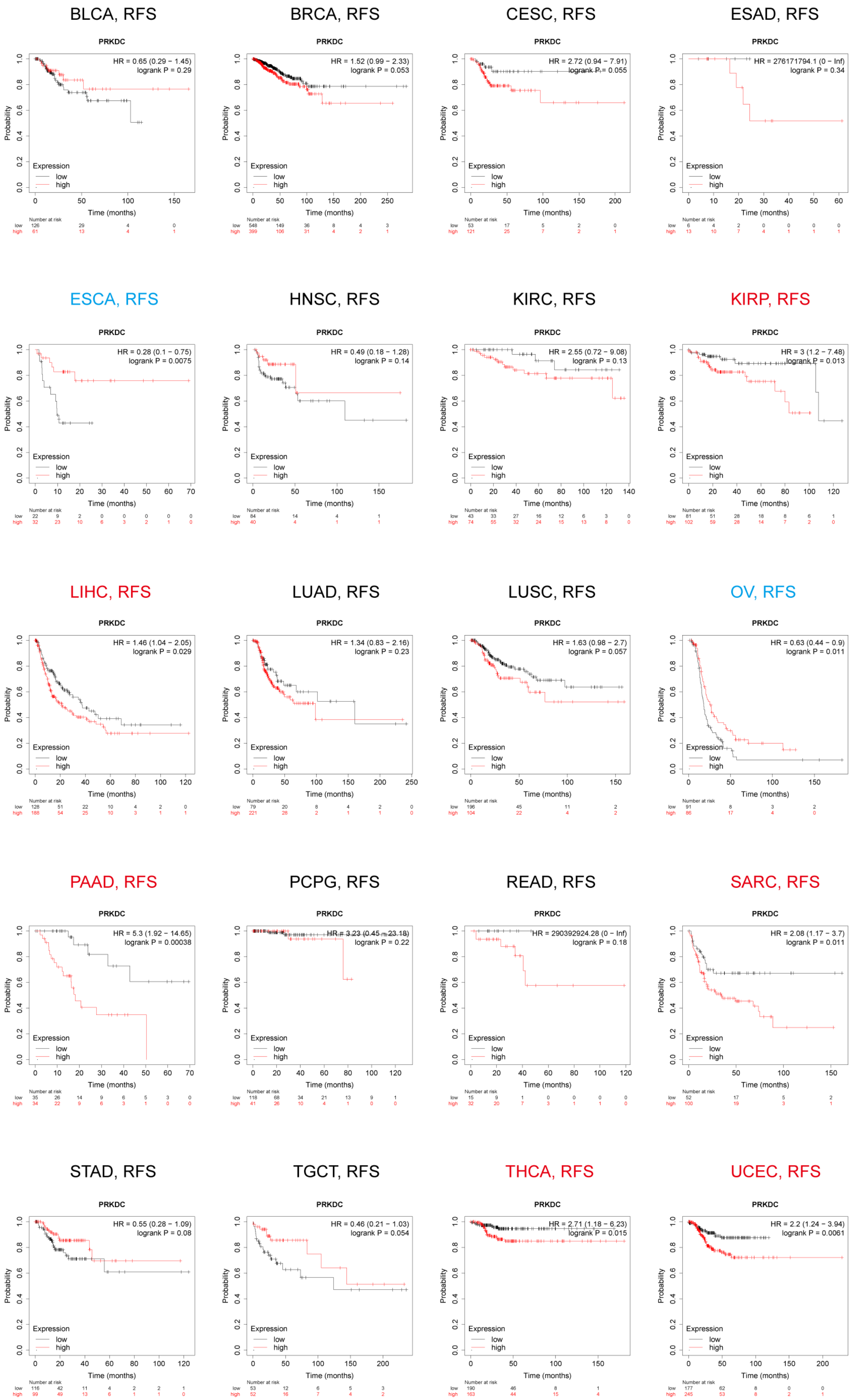
BLCA, RFS
PRKDC
HR = 0.65 (0.29 − 1.45)
logrank P = 0.29
BRCA, RFS
PRKDC
HR = 1.52 (0.99 − 2.33)
logrank P = 0.053
CESC, RFS
PRKDC
HR = 2.72 (0.94 − 7.91)
logrank P = 0.055
ESAD, RFS
PRKDC
HR = 276171794.1 (0 − Inf)
logrank P = 0.34
ESCA, RFS
PRKDC
HR = 0.28 (0.1 − 0.75)
logrank P = 0.0075
HNSC, RFS
PRKDC
HR = 0.49 (0.18 − 1.28)
logrank P = 0.14
KIRC, RFS
PRKDC
HR = 2.55 (0.72 − 9.08)
logrank P = 0.13
KIRP, RFS
PRKDC
HR = 3 (1.2 − 7.48)
logrank P = 0.013
LIHC, RFS
PRKDC
HR = 1.46 (1.04 − 2.05)
logrank P = 0.029
LUAD, RFS
PRKDC
HR = 1.34 (0.83 − 2.16)
logrank P = 0.23
LUSC, RFS
PRKDC
HR = 1.63 (0.98 − 2.7)
logrank P = 0.057
OV, RFS
PRKDC
HR = 0.63 (0.44 − 0.9)
logrank P = 0.011
PAAD, RFS
PRKDC
HR = 5.3 (1.92 − 14.65)
logrank P = 0.00038
PCPG, RFS
PRKDC
HR = 3.23 (0.45 − 23.18)
logrank P = 0.22
READ, RFS
PRKDC
HR = 290392924.28 (0 − Inf)
logrank P = 0.18
SARC, RFS
PRKDC
HR = 2.08 (1.17 − 3.7)
logrank P = 0.011
STAD, RFS
PRKDC
HR = 0.55 (0.28 − 1.09)
logrank P = 0.08
TGCT, RFS
PRKDC
HR = 0.46 (0.21 − 1.03)
logrank P = 0.054
THCA, RFS
PRKDC
HR = 2.71 (1.18 − 6.23)
logrank P = 0.015
UCEC, RFS
PRKDC
HR = 2.2 (1.24 − 3.94)
logrank P = 0.0061
Probability
Time (months)
Expression
low
high
Number at risk

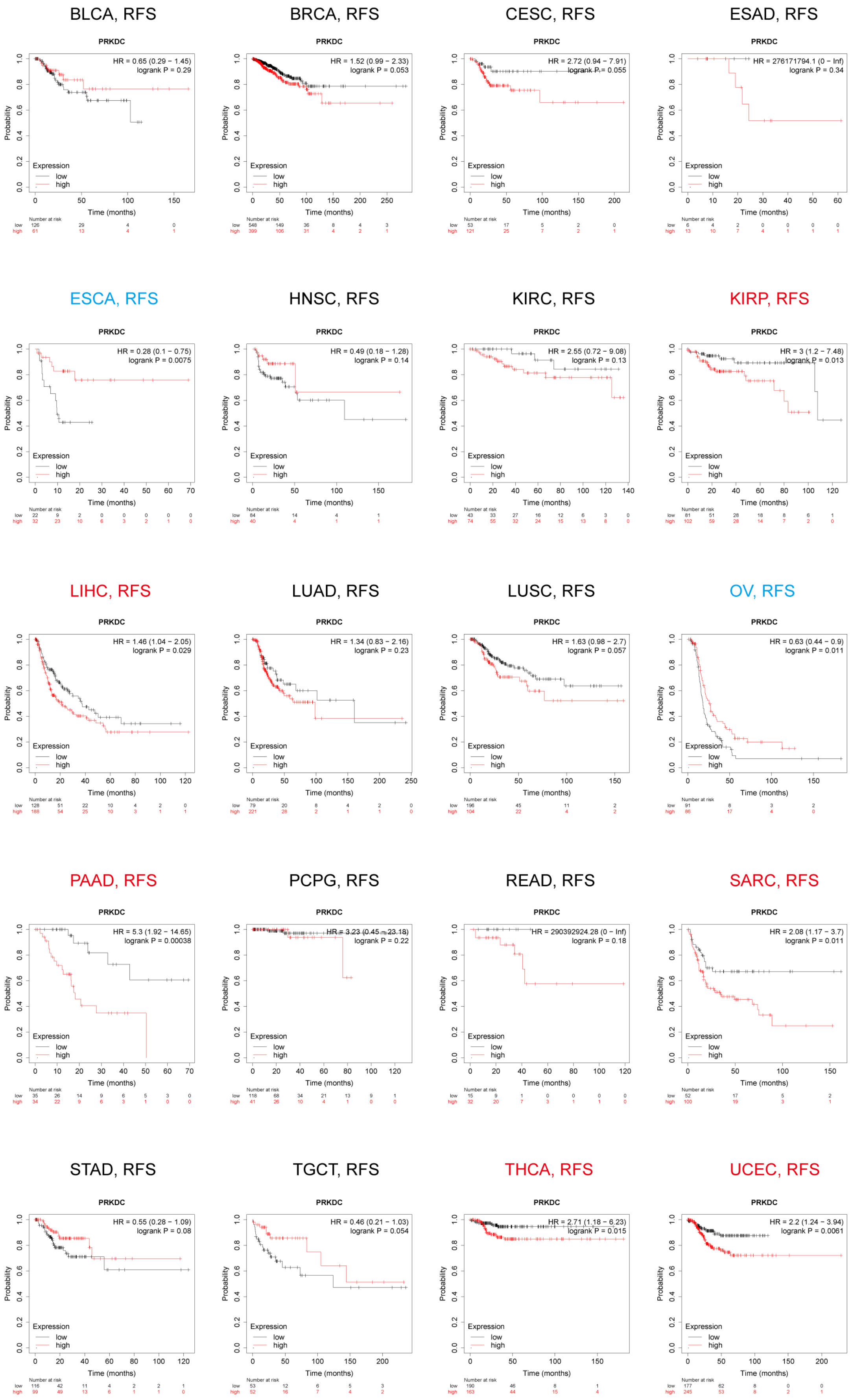
BLCA, RFS
PRKDC
HR = 0.65 (0.29 − 1.45)
logrank P = 0.29
BRCA, RFS
PRKDC
HR = 1.52 (0.99 − 2.33)
logrank P = 0.053
CESC, RFS
PRKDC
HR = 2.72 (0.94 − 7.91)
logrank P = 0.055
ESAD, RFS
PRKDC
HR = 276171794.1 (0 − Inf)
logrank P = 0.34
ESCA, RFS
PRKDC
HR = 0.28 (0.1 − 0.75)
logrank P = 0.0075
HNSC, RFS
PRKDC
HR = 0.49 (0.18 − 1.28)
logrank P = 0.14
KIRC, RFS
PRKDC
HR = 2.55 (0.72 − 9.08)
logrank P = 0.13
KIRP, RFS
PRKDC
HR = 3 (1.2 − 7.48)
logrank P = 0.013
LIHC, RFS
PRKDC
HR = 1.46 (1.04 − 2.05)
logrank P = 0.029
LUAD, RFS
PRKDC
HR = 1.34 (0.83 − 2.16)
logrank P = 0.23
LUSC, RFS
PRKDC
HR = 1.63 (0.98 − 2.7)
logrank P = 0.057
OV, RFS
PRKDC
HR = 0.63 (0.44 − 0.9)
logrank P = 0.011
PAAD, RFS
PRKDC
HR = 5.3 (1.92 − 14.65)
logrank P = 0.00038
PCPG, RFS
PRKDC
HR = 3.23 (0.45 − 23.18)
logrank P = 0.22
READ, RFS
PRKDC
HR = 290392924.28 (0 − Inf)
logrank P = 0.18
SARC, RFS
PRKDC
HR = 2.08 (1.17 − 3.7)
logrank P = 0.011
STAD, RFS
PRKDC
HR = 0.55 (0.28 − 1.09)
logrank P = 0.08
TGCT, RFS
PRKDC
HR = 0.46 (0.21 − 1.03)
logrank P = 0.054
THCA, RFS
PRKDC
HR = 2.71 (1.18 − 6.23)
logrank P = 0.015
UCEC, RFS
PRKDC
HR = 2.2 (1.24 − 3.94)
logrank P = 0.0061
Probability
Time (months)
Expression
low
high
Number at risk

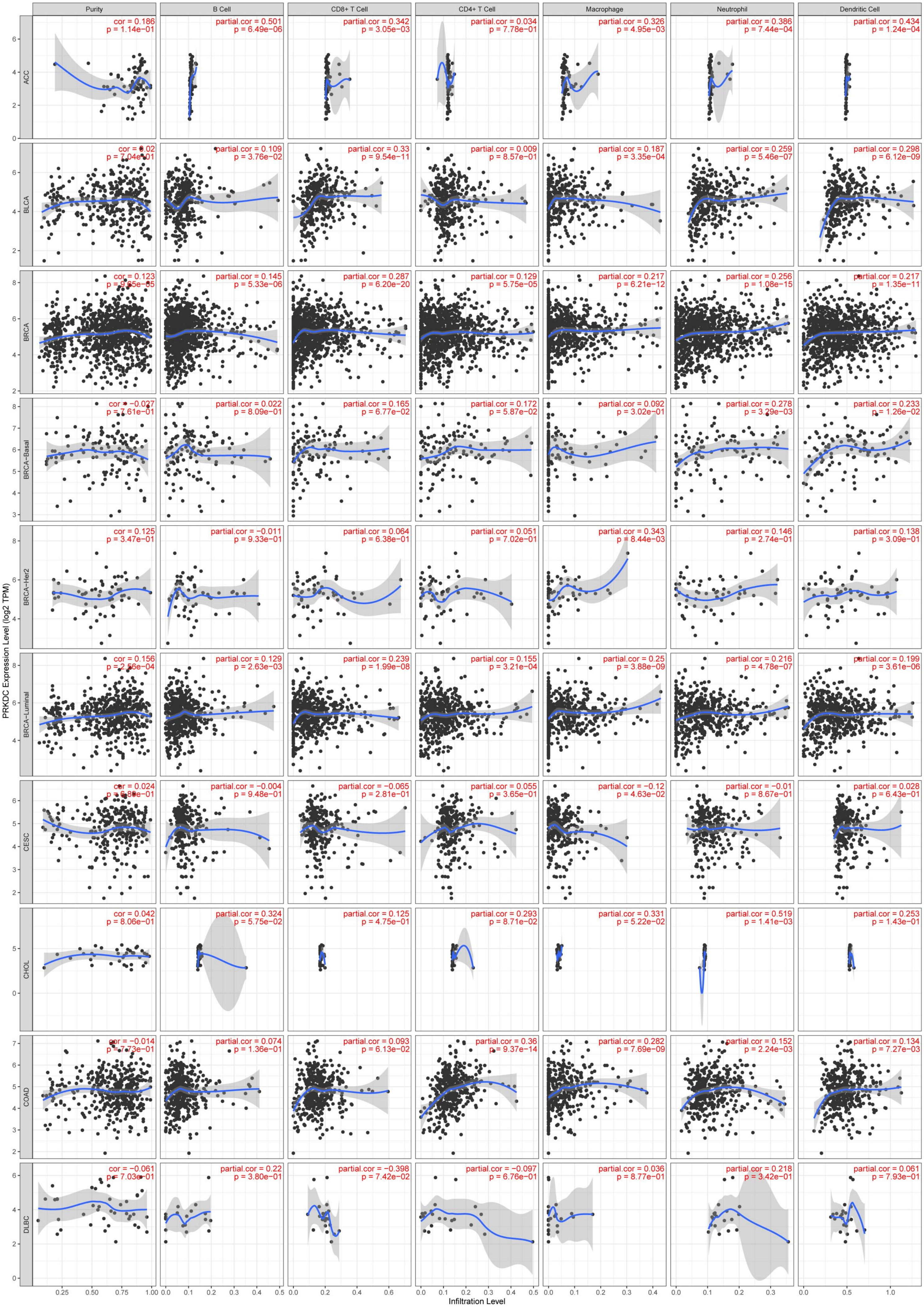

Purity
B Cell
CD8+ T Cell
CD4+ T Cell
Macrophage
Neutrophil
Dendritic Cell
ACC
cor = 0.186
p = 1.14e-01
partial.cor = 0.501
p = 6.49e-06
partial.cor = 0.342
p = 3.05e-03
partial.cor = 0.034
p = 7.78e-01
partial.cor = 0.326
p = 4.95e-03
partial.cor = 0.386
p = 7.44e-04
partial.cor = 0.434
p = 1.24e-04
BLCA
cor = 0.02
p = 7.04e-01
partial.cor = 0.109
p = 3.76e-02
partial.cor = 0.33
p = 9.54e-11
partial.cor = 0.009
p = 8.57e-01
partial.cor = 0.187
p = 3.35e-04
partial.cor = 0.259
p = 5.46e-07
partial.cor = 0.298
p = 6.12e-09
BRCA
cor = 0.123
p = 9.65e-05
partial.cor = 0.145
p = 5.33e-06
partial.cor = 0.287
p = 6.20e-20
partial.cor = 0.129
p = 5.75e-05
partial.cor = 0.217
p = 6.21e-12
partial.cor = 0.256
p = 1.08e-15
partial.cor = 0.217
p = 1.35e-11
BRCA−Basal
cor = −0.027
p = 7.61e-01
partial.cor = 0.022
p = 8.09e-01
partial.cor = 0.165
p = 6.77e-02
partial.cor = 0.172
p = 5.87e-02
partial.cor = 0.092
p = 3.02e-01
partial.cor = 0.278
p = 3.29e-03
partial.cor = 0.233
p = 1.26e-02
BRCA−Her2
cor = 0.125
p = 3.47e-01
partial.cor = −0.011
p = 9.33e-01
partial.cor = 0.064
p = 6.38e-01
partial.cor = 0.051
p = 7.02e-01
partial.cor = 0.343
p = 8.44e-03
partial.cor = 0.146
p = 2.74e-01
partial.cor = 0.138
p = 3.09e-01
BRCA−Luminal
cor = 0.156
p = 2.56e-04
partial.cor = 0.129
p = 2.63e-03
partial.cor = 0.239
p = 1.99e-08
partial.cor = 0.155
p = 3.21e-04
partial.cor = 0.25
p = 3.88e-09
partial.cor = 0.216
p = 4.78e-07
partial.cor = 0.199
p = 3.61e-06
CESC
cor = 0.024
p = 6.89e-01
partial.cor = −0.004
p = 9.48e-01
partial.cor = −0.065
p = 2.81e-01
partial.cor = 0.055
p = 3.65e-01
partial.cor = −0.12
p = 4.63e-02
partial.cor = −0.01
p = 8.67e-01
partial.cor = 0.028
p = 6.43e-01
CHOL
cor = 0.042
p = 8.06e-01
partial.cor = 0.324
p = 5.75e-02
partial.cor = 0.125
p = 4.75e-01
partial.cor = 0.293
p = 8.71e-02
partial.cor = 0.331
p = 5.22e-02
partial.cor = 0.519
p = 1.41e-03
partial.cor = 0.253
p = 1.43e-01
COAD
cor = −0.014
p = 7.73e-01
partial.cor = 0.074
p = 1.36e-01
partial.cor = 0.093
p = 6.13e-02
partial.cor = 0.36
p = 9.37e-14
partial.cor = 0.282
p = 7.69e-09
partial.cor = 0.152
p = 2.24e-03
partial.cor = 0.134
p = 7.27e-03
DLBC
cor = −0.061
p = 7.03e-01
partial.cor = 0.22
p = 3.80e-01
partial.cor = −0.398
p = 7.42e-02
partial.cor = −0.097
p = 6.76e-01
partial.cor = 0.036
p = 8.77e-01
partial.cor = 0.218
p = 3.42e-01
partial.cor = 0.061
p = 7.93e-01
PRKDC Expression Level (log2 TPM)
Infiltration Level

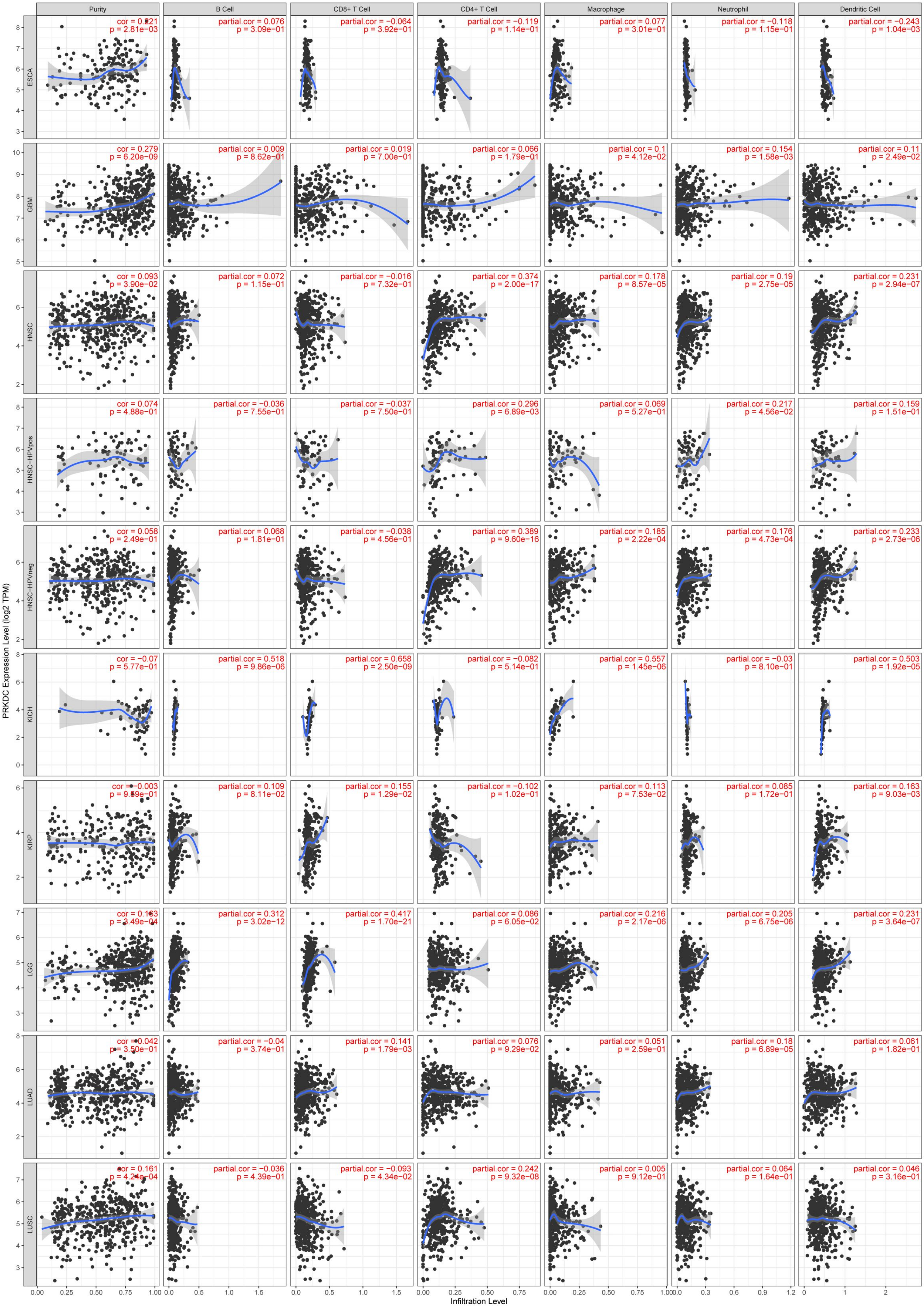

Purity
B Cell
CD8+ T Cell
CD4+ T Cell
Macrophage
Neutrophil
Dendritic Cell
ESCA
cor = 0.221
p = 2.81e-03
partial.cor = 0.076
p = 3.09e-01
partial.cor = −0.064
p = 3.92e-01
partial.cor = −0.119
p = 1.14e-01
partial.cor = 0.077
p = 3.01e-01
partial.cor = −0.118
p = 1.15e-01
partial.cor = −0.243
p = 1.04e-03
GBM
cor = 0.279
p = 6.20e-09
partial.cor = 0.009
p = 8.62e-01
partial.cor = 0.019
p = 7.00e-01
partial.cor = 0.066
p = 1.79e-01
partial.cor = 0.1
p = 4.12e-02
partial.cor = 0.154
p = 1.58e-03
partial.cor = 0.11
p = 2.49e-02
HNSC
cor = 0.093
p = 3.90e-02
partial.cor = 0.072
p = 1.15e-01
partial.cor = −0.016
p = 7.32e-01
partial.cor = 0.374
p = 2.00e-17
partial.cor = 0.178
p = 8.57e-05
partial.cor = 0.19
p = 2.75e-05
partial.cor = 0.231
p = 2.94e-07
HNSC−HPVpos
cor = 0.074
p = 4.88e-01
partial.cor = −0.036
p = 7.55e-01
partial.cor = −0.037
p = 7.50e-01
partial.cor = 0.296
p = 6.89e-03
partial.cor = 0.069
p = 5.27e-01
partial.cor = 0.217
p = 4.56e-02
partial.cor = 0.159
p = 1.51e-01
HNSC−HPVneg
cor = 0.058
p = 2.49e-01
partial.cor = 0.068
p = 1.81e-01
partial.cor = −0.038
p = 4.56e-01
partial.cor = 0.389
p = 9.60e-16
partial.cor = 0.185
p = 2.22e-04
partial.cor = 0.176
p = 4.73e-04
partial.cor = 0.233
p = 2.73e-06
KICH
cor = −0.07
p = 5.77e-01
partial.cor = 0.518
p = 9.86e-06
partial.cor = 0.658
p = 2.50e-09
partial.cor = −0.082
p = 5.14e-01
partial.cor = 0.557
p = 1.45e-06
partial.cor = −0.03
p = 8.10e-01
partial.cor = 0.503
p = 1.92e-05
KIRP
cor = −0.003
partial.cor = 0.109
p = 8.11e-02
partial.cor = 0.155
p = 1.29e-02
partial.cor = −0.102
p = 1.02e-01
partial.cor = 0.113
p = 7.53e-02
partial.cor = 0.085
p = 1.72e-01
partial.cor = 0.163
p = 9.03e-03
LGG
p = 3.49e-04
partial.cor = 0.312
p = 3.02e-12
partial.cor = 0.417
p = 1.70e-21
partial.cor = 0.086
p = 6.05e-02
partial.cor = 0.216
p = 2.17e-06
partial.cor = 0.205
p = 6.75e-06
partial.cor = 0.231
p = 3.64e-07
LUAD
cor = −0.042
p = 3.50e-01
partial.cor = −0.04
p = 3.74e-01
partial.cor = 0.141
p = 1.79e-03
partial.cor = 0.076
p = 9.29e-02
partial.cor = 0.051
p = 2.59e-01
partial.cor = 0.18
p = 6.89e-05
partial.cor = 0.061
p = 1.82e-01
LUSC
cor = 0.161
partial.cor = −0.036
p = 4.39e-01
partial.cor = −0.093
p = 4.34e-02
partial.cor = 0.242
p = 9.32e-08
partial.cor = 0.005
p = 9.12e-01
partial.cor = 0.064
p = 1.64e-01
partial.cor = 0.046
p = 3.16e-01
PRKDC Expression Level (log2 TPM)
Infiltration Level

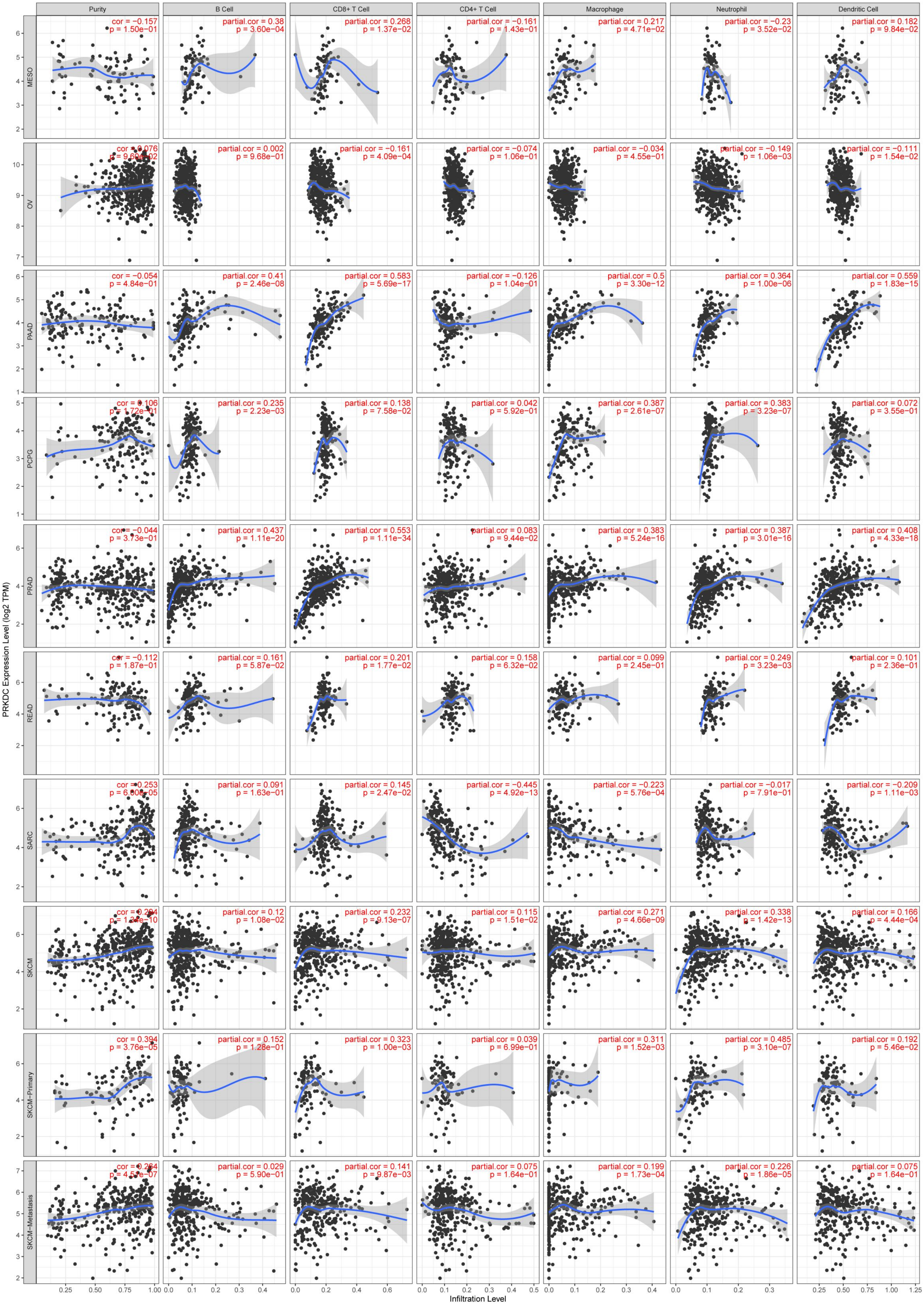

Purity
B Cell
CD8+ T Cell
CD4+ T Cell
Macrophage
Neutrophil
Dendritic Cell
MESO
cor = −0.157 p = 1.50e−01
partial.cor = 0.38 p = 3.60e−04
partial.cor = 0.268 p = 1.37e−02
partial.cor = −0.161 p = 1.43e−01
partial.cor = 0.217 p = 4.71e−02
partial.cor = −0.23 p = 3.52e−02
partial.cor = 0.182 p = 9.84e−02
OV
partial.cor = 0.002 p = 9.68e−01
partial.cor = −0.161 p = 4.09e−04
partial.cor = −0.074 p = 1.06e−01
partial.cor = −0.034 p = 4.55e−01
partial.cor = −0.149 p = 1.06e−03
partial.cor = −0.111 p = 1.54e−02
PAAD
cor = −0.054 p = 4.84e−01
partial.cor = 0.41 p = 2.46e−08
partial.cor = 0.583 p = 5.69e−17
partial.cor = −0.126 p = 1.04e−01
partial.cor = 0.5 p = 3.30e−12
partial.cor = 0.364 p = 1.00e−06
partial.cor = 0.559 p = 1.83e−15
PCPG
cor = 0.106 p = 1.72e−01
partial.cor = 0.235 p = 2.23e−03
partial.cor = 0.138 p = 7.58e−02
partial.cor = 0.042 p = 5.92e−01
partial.cor = 0.387 p = 2.61e−07
partial.cor = 0.383 p = 3.23e−07
partial.cor = 0.072 p = 3.55e−01
PRAD
cor = −0.044 p = 3.73e−01
partial.cor = 0.437 p = 1.11e−20
partial.cor = 0.553 p = 1.11e−34
partial.cor = 0.083 p = 9.44e−02
partial.cor = 0.383 p = 5.24e−16
partial.cor = 0.387 p = 3.01e−16
partial.cor = 0.408 p = 4.33e−18
READ
cor = −0.112 p = 1.87e−01
partial.cor = 0.161 p = 5.87e−02
partial.cor = 0.201 p = 1.77e−02
partial.cor = 0.158 p = 6.32e−02
partial.cor = 0.099 p = 2.45e−01
partial.cor = 0.249 p = 3.23e−03
partial.cor = 0.101 p = 2.36e−01
SARC
partial.cor = 0.091 p = 1.63e−01
partial.cor = 0.145 p = 2.47e−02
partial.cor = −0.445 p = 4.92e−13
partial.cor = −0.223 p = 5.76e−04
partial.cor = −0.017 p = 7.91e−01
partial.cor = −0.209 p = 1.11e−03
SKCM
partial.cor = 0.12 p = 1.08e−02
partial.cor = 0.232 p = 9.13e−07
partial.cor = 0.115 p = 1.51e−02
partial.cor = 0.271 p = 4.66e−09
partial.cor = 0.338 p = 1.42e−13
partial.cor = 0.166 p = 4.44e−04
SKCM−Primary
cor = 0.394 p = 3.76e−05
partial.cor = 0.152 p = 1.28e−01
partial.cor = 0.323 p = 1.00e−03
partial.cor = 0.039 p = 6.99e−01
partial.cor = 0.311 p = 1.52e−03
partial.cor = 0.485 p = 3.10e−07
partial.cor = 0.192 p = 5.46e−02
SKCM−Metastasis
partial.cor = 0.029 p = 5.90e−01
partial.cor = 0.141 p = 9.87e−03
partial.cor = 0.075 p = 1.64e−01
partial.cor = 0.199 p = 1.73e−04
partial.cor = 0.226 p = 1.86e−05
partial.cor = 0.075 p = 1.64e−01
PRKDC Expression Level (log2 TPM)
Infiltration Level

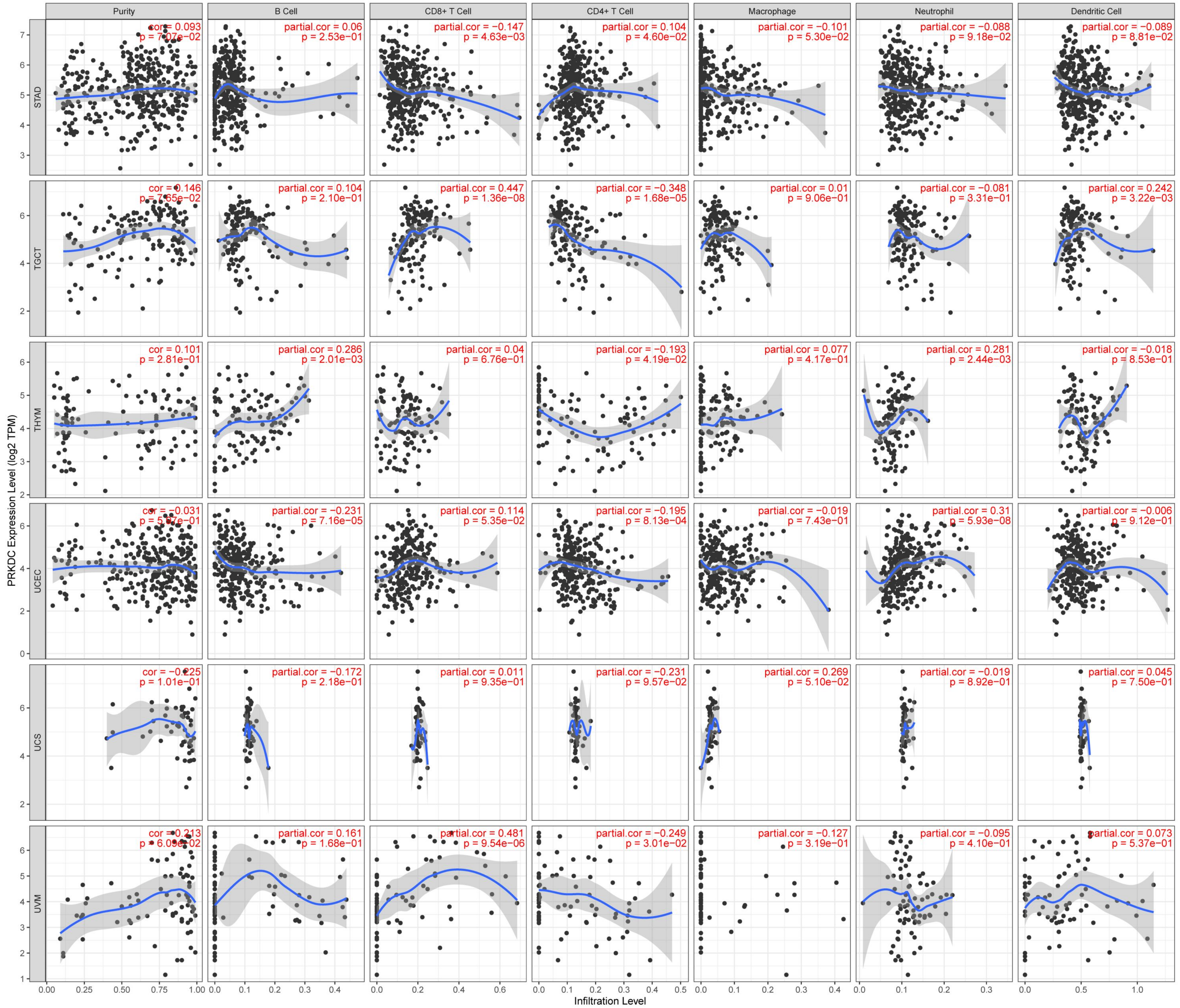
Purity
B Cell
CD8+ T Cell
CD4+ T Cell
Macrophage
Neutrophil
Dendritic Cell
STAD
TGCT
THYM
UCEC
UCS
UVM
PRKDC Expression Level (log2 TPM)
Infiltration Level
cor = 0.093 p = 7.07e−02
partial.cor = 0.06 p = 2.53e−01
partial.cor = −0.147 p = 4.63e−03
partial.cor = 0.104 p = 4.60e−02
partial.cor = −0.101 p = 5.30e−02
partial.cor = −0.088 p = 9.18e−02
partial.cor = −0.089 p = 8.81e−02
cor = 0.146 p = 7.65e−02
partial.cor = 0.104 p = 2.10e−01
partial.cor = 0.447 p = 1.36e−08
partial.cor = −0.348 p = 1.68e−05
partial.cor = 0.01 p = 9.06e−01
partial.cor = −0.081 p = 3.31e−01
partial.cor = 0.242 p = 3.22e−03
cor = 0.101 p = 2.81e−01
partial.cor = 0.286 p = 2.01e−03
partial.cor = 0.04 p = 6.76e−01
partial.cor = −0.193 p = 4.19e−02
partial.cor = 0.077 p = 4.17e−01
partial.cor = 0.281 p = 2.44e−03
partial.cor = −0.018 p = 8.53e−01
cor = −0.031 p = 5.87e−01
partial.cor = −0.231 p = 7.16e−05
partial.cor = 0.114 p = 5.35e−02
partial.cor = −0.195 p = 8.13e−04
partial.cor = −0.019 p = 7.43e−01
partial.cor = 0.31 p = 5.93e−08
partial.cor = −0.006 p = 9.12e−01
cor = −0.225 p = 1.01e−01
partial.cor = −0.172 p = 2.18e−01
partial.cor = 0.011 p = 9.35e−01
partial.cor = −0.231 p = 9.57e−02
partial.cor = 0.269 p = 5.10e−02
partial.cor = −0.019 p = 8.92e−01
partial.cor = 0.045 p = 7.50e−01
cor = 0.213 p = 6.09e−02
partial.cor = 0.161 p = 1.68e−01
partial.cor = 0.481 p = 9.54e−06
partial.cor = −0.249 p = 3.01e−02
partial.cor = −0.127 p = 3.19e−01
partial.cor = −0.095 p = 4.10e−01
partial.cor = 0.073 p = 5.37e−01
